# An improved understanding of ungulate population dynamics using count data: Insights from western Montana

**DOI:** 10.1371/journal.pone.0226492

**Published:** 2019-12-23

**Authors:** J. Terrill Paterson, Kelly Proffitt, Jay Rotella, Robert Garrott

**Affiliations:** 1 Department of Ecology, Montana State University, Bozeman, Montana, United States of America; 2 Montana Department of Fish, Wildlife and Parks, Bozeman, Montana, United States of America; National Oceanic and Atmospheric Administration, UNITED STATES

## Abstract

Understanding the dynamics of ungulate populations is critical given their ecological and economic importance. In particular, the ability to evaluate the evidence for potential drivers of variation in population trajectories is important for informed management. However, the use of age ratio data (e.g., juveniles:adult females) as an index of variation in population dynamics is hindered by a lack of statistical power and difficult interpretation. Here, we show that the use of a population model based on count, classification and harvest data can dramatically improve the understanding of ungulate population dynamics by: 1) providing estimates of vital rates (e.g., per capita recruitment and population growth) that are easier to interpret and more useful to managers than age ratios and 2) increasing the power to assess potential sources of variation in key vital rates. We used a time series of elk (*Cervus canadensis*) spring count and classification data (2004 to 2016) and fall harvest data from hunting districts in western Montana to construct a population model to estimate vital rates and assess evidence for an association between a series of environmental covariates and indices of predator abundance on per capita recruitment rates of elk calves. Our results suggest that per capita recruitment rates were negatively associated with cold and wet springs, and severe winters, and positively associated with summer precipitation. In contrast, an analysis of the raw age ratio data failed to detect these relationships. Our approach based on a population model provided estimates of the region-wide mean per capita recruitment rate (mean = 0.25, 90% CI = 0.21, 0.29), temporal variation in hunting-district-specific recruitment rates (minimum = 0.09; 90% CI = [0.07, 0.11], maximum = 0.43; 90% CI = [0.38, 0.48]), and annual population growth rates (minimum = 0.83; 90% CI = [0.78, 0.87], maximum = 1.20; 90% CI = [1.11, 1.29]). We recommend using routinely collected population count and classification data and a population modeling approach rather than interpreting estimated age ratios as a substantial improvement in understanding population dynamics.

## Introduction

The population dynamics of ungulates reflect complicated interactions between abiotic and biotic factors such as environmental variation, predation and harvest [1–3]. Understanding the relative influence of each of these factors on population dynamics is critical given the pivotal role of ungulates in ecosystems [4] and concerns about declines in multiple ungulate populations [5–7]. Populations of ungulates face challenges due to broad-scale habitat changes [8], climate change and asynchrony of phenological patterns [5], over-harvest [9], and the restoration of predator communities [10]. The management of ungulate populations takes place in the midst of considerable uncertainty as to the relative influence of these factors on populations, compounded by uncertainty over the degree to which management actions can affect them [11,12].

The trajectories of populations through time are the integrated result of a group of co-varying vital rates (e.g., survival, reproduction, recruitment), and effective management requires the identification of those rates responsible for demographic performance [13]. Although variation in adult female survival rates has the highest proportional impact on population growth rate, theoretical and empirical work strongly suggest that adult survival rates are buffered against high variation [14–17]. In contrast, juvenile survival can have a large impact on population growth rates when interannual variation is large [18–20]. Thus, juvenile survival is commonly monitored and used as an index of population performance. However, juvenile survival varies annually, and causes of mortality differ widely across ecosystems [19,21], making it difficult to understand and generalize conclusions about sources of variation in juvenile survival.

Given the practical challenges of studies on individually marked animals, many ungulate populations are routinely monitored and managed using observed age ratios (e.g., juveniles per 100 adult females) as a proxy for juvenile survival [19,22]. In contrast to data on individually marked individuals, data on age ratios are comparatively easy to acquire and widely applicable to management of multiple species, which has led to routine collection of age ratio data and the development of long time series of ratios [22–24]. However, population management decisions informed by age ratios are challenged because they conflate variation in two age classes and distill complicated population dynamics into a single summary statistic [23–26]. Moreover, the interpretation of age ratios from harvested populations of ungulates can be further complicated by the timing of surveys relative to harvest. For age ratio data collected in the spring, the numerator (juveniles) is driven by rates of pregnancy and calf survival from birth to the time of the count, whereas the denominator (counts of adult females) is driven by adult survival and harvest. Thus, the harvest the previous fall can drive variation in age ratios by reducing the counts of adult females.

In addition to the challenges of using age ratios as indices of demographic performance, a separate challenge is posed by their use as a response variable in the log-linear or linear models that are typically used to evaluate sources of variation in population dynamics [27,28]. Perfectly observed age ratios should reflect process variance, or variation that is the result of stochasticity in the underlying time series of biological processes such as conception rates, juvenile survival, adult survival and harvest. However, imperfect observation of individuals during surveys introduces an additional source of error such that observed age ratio data combine both process variance and observation error. Consequently, they are a noisy signal into underlying population dynamics [27,29]. This conflation of errors significantly reduces the power of linear or log-linear models to detect sources of variation in vital rates [30,31].

An alternative modeling approach uses a state-space approach to connect the counts of animals through time using a population model that explicitly incorporates key biological processes while separately modeling the observation process, rather than distilling the counts to a single ratio [27,30,32]. The state-space approach, wherein the latent states are the unobserved population abundances driven by reproduction, survival and harvest, has been shown to dramatically improve biological inference into sources of variation in vital rates even if the population model is mis-specified [30,31,33,34]. Moreover, these models allow inference about population trajectories and growth rates when the observed abundance is biased due to imperfect detection, provided that the observed population is a reliable index to the larger population [35]. Additionally, a Bayesian formulation of the state-space approach can accommodate situations where data are missing and/or classification errors exist for some age/sex classes [35,36]. Importantly, this modeling approach uses data that are already routinely collected by wildlife managers, i.e., the numbers of individuals observed in each class, to make inference about the key vital rate for which age ratios are a proxy: the per capita recruitment rate.

**Fig 1:**
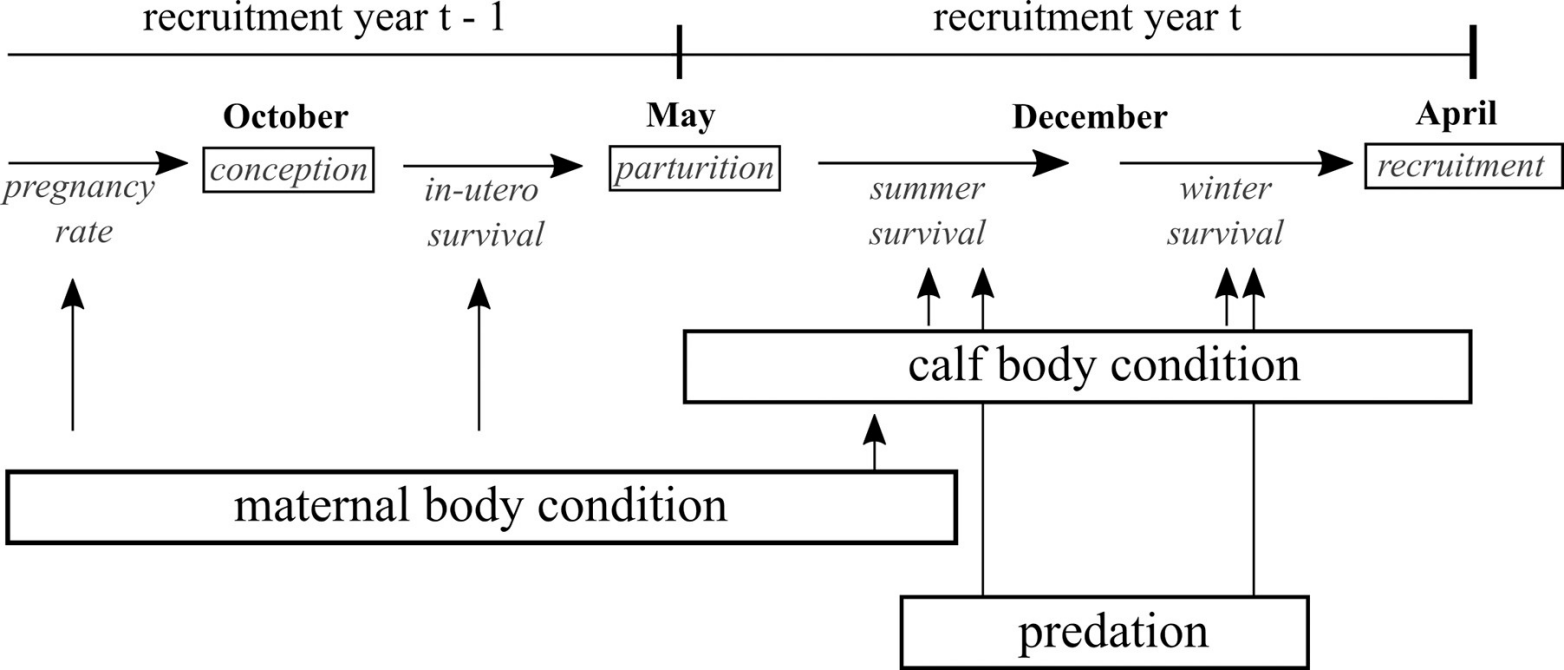
Concept diagram illustrating the progression of events and rates underlying recruitment for spring surveys. The probability that each calf is available to be surveyed during the late spring surveys is the product of pregnancy rates, in-utero survival to parturition, then summer and winter survival, the product of which is the per capita recruitment rate. Each rate is hypothesized to be associated with multiple drivers related to the timing of events.

The per capita recruitment rate can drive the population dynamics of ungulates and is the result of a series of processes that are potentially affected by environmental conditions and predator pressure (Fig 1). Maternal body condition from the summer prior to conception (year t-1) through parturition has been shown to be related to pregnancy rates [37,38], parturition mass [39] and neonatal survival during the maternal care period following birth [2]. Therefore, we expected per capita recruitment rates to be positively associated with indices of nutrition, negatively associated with winter severity (year t-1), and potentially demonstrate an interaction between nutrition indices and winter severity such that poor summer conditions and severe winter conditions combine to further reduce recruitment [40]. Environmental conditions experienced after parturition (year t) are thought to be related to juvenile survival in its first year, either through its direct impact on juvenile nutrition through foraging [41] or as mediated through maternal provisioning during the maternal care period [38]. There is an evolving debate as to whether spring conditions or late summer conditions are more important to juvenile survival [42], and we split indices of the nutritional environment into spring and summer periods to assess the relative importance of these two periods. We expected per capita recruitment rates to be positively associated with indices of nutrition (year t). Juvenile survival to recruitment has been shown to be related to winter conditions [43] and we expected per capita recruitment rates to be negatively associated with winter severity (year t), and interact with nutritional conditions such that the impact of poor nutritional conditions is made worse in severe winters. Predators can have a large impact on juvenile survival [3,10,21,44], and we expected per capita recruitment rates to be negatively associated with indices of predator abundance. Similar to nutritional conditions, empirical work has suggested that the impact of predation on ungulate populations can be affected by winter conditions [45,46], highlighting the need to investigate interactions between indices of predator abundance and winter severity.

Here, our goals were two-fold. First, we analyzed a time-series of data on harvested elk (*Cervus canadensis*) herds in western Montana that contained spring count and fall harvest data to assess the strength of evidence for a variety of potential sources of variation in recruitment rates using (1) a state-space approach (hereafter, the “population model”) and (2) a standard age ratio approach (“age ratio model”). Second, we compared results from the two models to evaluate and understand important differences in what could be learned from each analytical approach. We predicted the population model would provide more information on population growth rates and trajectories that can inform management (e.g., estimates of *λ*) as well as the observation processes.

## Methods

### Ethics statement

This was an observational study that relied on aerial count data and estimated harvest statistics from the Montana Department of Fish, Wildlife and Parks. No animals were handled and no private lands accessed in the course of this study.

### Study area

**Fig 2:**
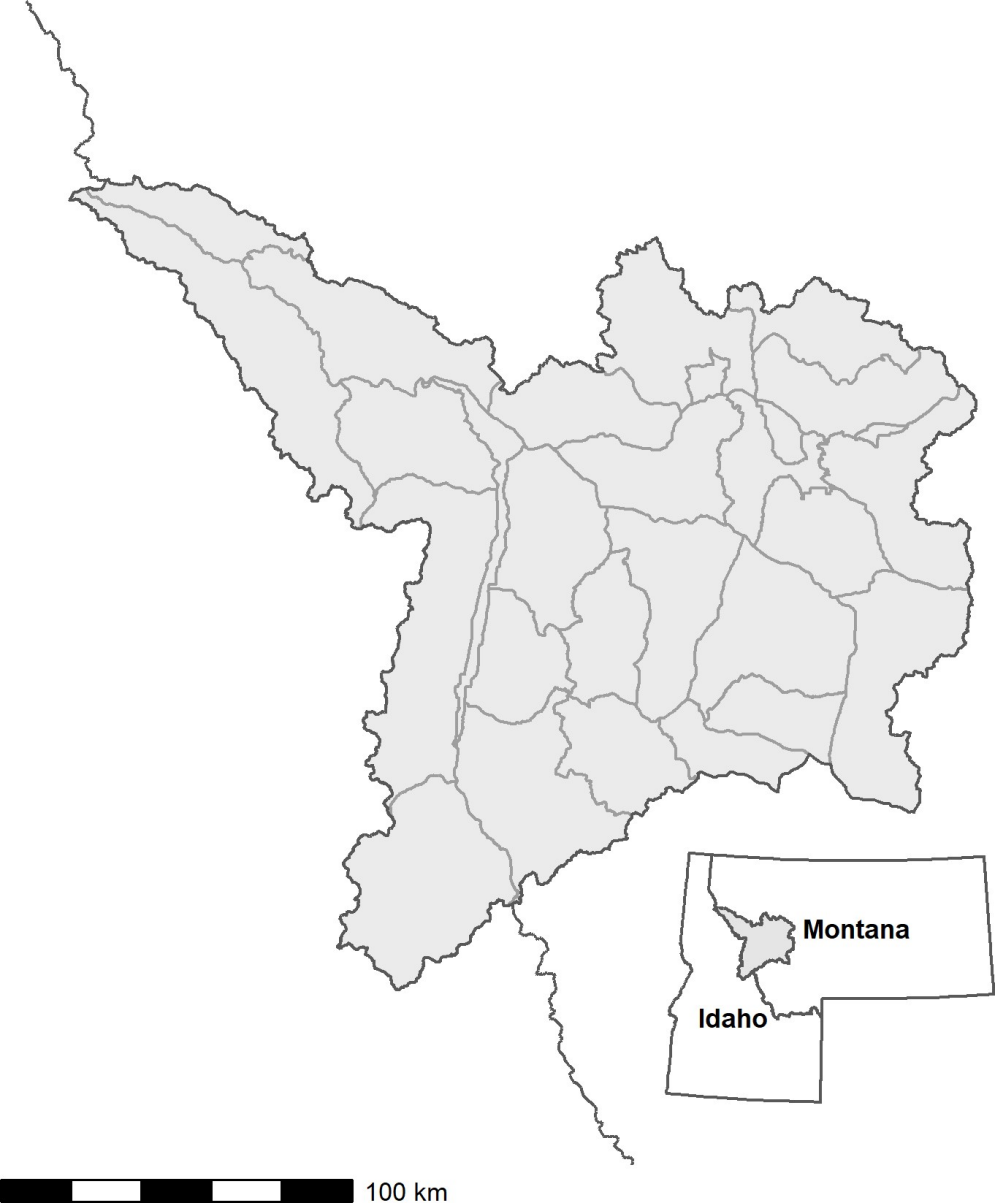
Study area in western Montana. The study area was comprised of 28 hunting districts within the administrative Region 2 management area. Data were used from 17 of the hunting districts.

Our 27,318 km^2^ study area contained 28 elk hunting districts in western Montana (46.0216° N, 114.1731° W) that were defined by the state wildlife agency (Montana Department of Fish, Wildlife and Parks) based on biological and logistical boundaries (Fig 2). The hunting districts vary in size from 44 km^2^ to 1,991 km^2^ (mean = 976 km^2^, sd = 516 km^2^) and are distributed across a series of physiographic gradients. Elevation ranges across the study area from 767 m to 3,200 m, with a mean within-district range of 976 m (sd = 516 m). The terrain ranges from flat (minimum 100m slope variance = 0) to rugged (maximum 100m slope variance = 0.25). Over the 13-years of our study (2004 to 2016) precipitation in late spring (May to June) ranged from 40 mm to 568 mm (mean within-district range = 257.43 mm, sd = 100 mm), whereas winter precipitation from December to March ranged from 33 mm to 1,650 mm (mean within-district range = 677.2 mm, sd = 370 mm).

### Count data

We used annual spring elk count and age/sex classification data collected from fixed wing aircraft (March-April). Surveys were conducted annually on the winter range for each district in the late spring prior to the migration to summer range and the birth pulse. All surveys were conducted to minimize the potential for double counting, and most surveys were completed within a single day. Due to logistical limitations, not every district had count and age/sex classification data for each of the 13 years (2004 to 2016), which generated a discontinuous time series for most districts (median number of years = 7, minimum = 6, maximum = 13). Age ratios were derived from the total counts from the surveys. In our analysis, we included all hunting districts that had a minimum of 6 years of count data collected during 2004 to 2016 (17 management districts, S1 Fig). For a small number of district-years a total count was available, but no age/sex classification was reported (n = 5). For the population modeling approach, we were able to treat the age/sex classifications in these years as missing data, which was not possible in the age ratio approach. This resulted in two different-sized data sets: 1) the age ratio model had 135 district-years of observed age ratios, and 2) the population model approach had 140 district-years of count data. Moreover, in each district-year not all of the animals that were counted were subsequently classified according to age/sex class, i.e., the number of animals in each age and sex classification represented a sample of the total number of animals that were counted.

### Model descriptions

#### Population model

The population model approach linked two separate processes: 1) a model for the biological processes of elk survival, recruitment and harvest, and 2) the observation process that gave rise to data.

Similar to previous work in this system [20], we defined the annual population cycle from the birth pulse (in May-June) to the following spring (March-April) when calves recruit to the population as 1-year-olds. The population cycle can be represented as a stage-structured model, where the expected number (*E*) of calves (*N*^*c*^), adult females (the combination of yearlings and older females, *N*^af^) and adult males (the combination of yearlings and older males, *N*^am^) in year *t* and district *u* is given as:

$$E\left\{\begin{array}{c} N_{t,u}^{c}\\ N_{t,u}^{af}\\ N_{t,u}^{am} \end{array}\right\}=\left\{\begin{array}{c} {\tau N}_{t-1,u}^{af}-h_{t,u}^{c}\\ \phi_{a}(\delta {N_{t-1,u}^{c}+N}_{t-1,u}^{af}-h_{t,u}^{af})\\ \phi_{a}\left((1-\delta ){N_{t-1,u}^{c}+N}_{t-1,u}^{am}-h_{t,u}^{am}\right) \end{array}\right\}$$ (1)

where the vital rates that connect the population size across years are apparent adult survival (*ϕ*_*a*_), the proportion of calves that were female (*δ*, here assumed to be equal to 0.5), and the per capita recruitment rate (*τ*), and *h*^c^, *h*^af^, and *h*^am^ are age/sex specific harvest. The timing of the surveys (late spring) did not match the model anniversary (June 1), therefore the survival terms for calves represented an approximate 10-month survival probability. Individuals that were harvested in year *t* were subtracted from the population total in year *t-*1, i.e., treated as if harvest occurred instantaneously after surveys in late spring. Adult survival terms therefore represented survival after accounting for harvest. We assumed the survival of all age/sex classes other than calves was the same through time, a simplification given the evidence for age-related changes in adult survival in ungulates[47]. However, in managed populations variation in adult survival is thought to be driven by harvest rates [3,48] that are explicitly accounted for in our model, rendering the simplification plausible. Per capita recruitment is the product of a series of vital rates, including the probability of conception, in-utero survival to birth, and calf survival from birth to census the next spring. By construction, these vital rates were not separately identifiable.

Similar to previous work in this system [20], we used a Poisson distribution to incorporate demographic stochasticity, e.g., the number of adult females in year *t* and district *u* is a realization from a Poisson process with mean equal to the expected number from the matrix model above:

$$N_{t,u}^{af}∼Poisson\left(\phi_{a}(\delta N_{t-1,u}^{c}+N_{t-1,u}^{af}-h_{t,u}^{af})\right)$$ (2)

and the number of calves in year *t* and district *u* is:

$$N_{t,u}^{c}∼Poisson({\tau N}_{t-1,u}^{af}-h_{t,u}^{c}).$$ (3)

The observation process needed to accommodate cases wherein a total number of animals was counted, but only a sample of that total was then classified according to age and sex. To accommodate this data structure, we first modeled the total count of all elk as an overdispersed Poisson random variable with the mean equal to the unknown (latent) true population size:

$${Count}_{t,u}^{total}∼Poisson(N_{t,u}^{total}\gamma_{t,u})$$ (4)

where $N_{t,u}^{total}=N_{t,u}^{am}+N_{t,u}^{af}+N_{t,u}^{calves}$, and *γ*_*t*,*u*_ is an observation-level random effect at the unit-year level to accommodate potential overdispersion that would otherwise handicap the use of a Poisson [49]: $\gamma_{t,u}∼Normal(0,\sigma_{count}^{2})$, where the variance term is estimated by the model. An alternative approach would have been to model the counts using a negative binomial distribution. In our case, the biological inference was virtually identical to that from the Poisson model, and we have retained its use below. Notably, we have combined an observation process (aerial surveys) that is thought to typically be an underestimate of the true population size with an unbiased population model incorporating harvest [50]. The relationship between aerial counts and the true population size (sightability) is typically unknown, and in the presence of imperfect detection, population models estimate an “abundance” that is an index to the larger, true population. However, inference on vital rates remains robust in the presence of imperfect detection when variation in the probability of detection is random [35]. Furthermore, recent work indicates that inference on vital rates based on these models is robust even when combining biased counts with an unbiased population model. Estimates of population growth rates and fecundity are minimally biased when the probability of detection is less than 1, although there is likely a lower limit to the probability of detection where counts are a poor reflection of population dynamics [51].

Next, we used a multinomial distribution to connect the total number of animals classified (Classified_*t*,*u*_) to the classified number of calves, adult females and adult males:

$${\left[{Count}^{c},{Count}^{af},{Count}^{am}\right]}_{t,u}∼Multinomial(\pi_{t,u},{Classified}_{t,u})$$ (5)

We further assume that the number of enumerated calves, adult females and adult males are proportional to their representation in the underlying population:

$$\pi_{t,u}={\left[\frac{N^{c}}{N^{total}},\frac{N^{af}}{N^{total}},\frac{N^{am}}{N^{total}}\right]}_{t,u}$$ (6)

The goal of the population model was to partition available data into a structure that connected observations to underlying biological processes, which facilitated the expression of the vital rate of interest, *τ*, or per capita recruitment. Our fundamental goal was to then understand sources of variation in this key rate, and we incorporated covariates by using a logit link (which assumed the rate of offspring production at the population level was between 0 and 1):

$$logit(\tau_{t,u})=\alpha +\zeta_{t}+x_{t,u}\beta$$ (7)

where *α* is a common intercept (and corresponds to mean recruitment on the logit scale), *ζ*_*t*_ are mean-zero random effects for year, **x**_*t*,*u*_ is the vector of covariates and **β** the regression coefficients (S1 File). This model structure constrains vital rates to be the same across all the herds for a given set of covariate values in the same year and assumes that the response of each herd to variation in covariate values is the same, i.e., differences in recruitment rates between herds arise only from differing covariate values. We considered this a reasonable model structure given that this management region was defined based on similar ranges of habitat conditions and we had no *a priori* reason to suspect that herds would respond differently to external drivers.

#### Age ratio model

To mimic traditional analyses of age ratio data, we modeled the observed calves:100 adult females for year *t* in district *u* using a linear model with a Gaussian error structure:

$${\frac{Calves}{100\;Cows}}_{t,u}∼Normal(\mu_{t,u},\sigma_{age\;ratio}^{2})$$ (8)

where the expected value was modeled using an identity link and a similar structure to the population model:

$$\mu_{t,u}=\alpha +\zeta_{t}+x_{t,u}\beta +\beta^{harvest}h_{t,u}^{af}$$ (9)

where, unlike in the population model, $\beta^{harvest}h_{t,u}^{af}$ is included to account for the relationship between the harvest of adult females and the age ratio [7] (S1 File). This model was a standard linear regression approach that used the index of recruitment (calves:100 adult females) as the response to estimate regression parameters and the error term ($\sigma_{age\;ratio}^{2}$).

### Covariates

Our primary goal was to assess the strength of evidence for a series of potential sources of variation in the recruitment of elk calves as mediated through maternal body condition, calf body condition and predation risk (Fig 1). We developed covariates to index environmental conditions during the growing season, primary productivity, winter severity, and predator abundances.

We extracted precipitation values for the study area through time from the parameter-elevation regression on independent slopes model (PRISM Climate Group, Oregon State University, http://prism.oregonstate.edu, accessed 11 September 2018) [52]. To evaluate the support for the relative importance of late spring/early summer precipitation versus summer/late summer precipitation we created two covariates by first summing (at the pixel level) precipitation values over the two periods (late spring/early summer (neonatal period): May 1 to June 30, and late summer/early fall (juvenile independence period): July 1 to September 30). We then took the mean of all the summed pixels over the summer range for each hunting district to represent average cumulative precipitation in a hunting district for both periods. We assumed that values of the normalized difference vegetation index (NDVI) derived from the moderate resolution imaging spectroradiometer (MODIS) Terra satellite represented primary production on the landscape, and served as a proxy for annual forage productivity [53]. We used the 8-day surface reflectance images with 250m resolution (MODIS product MOD09Q1) to calculate NDVI values on a per-pixel basis across the study area and through time (courtesy of the NASA Land Processes Distributed Active Archive Center (LP DAAC), USGS/Earth Resources Observation and Science (EROS) Center, Sioux Falls, South Dakota) [54]. We smoothed the annual time series of NDVI values for each pixel by first applying a running mean, then applied an iterative interpolation algorithm that adjusted values to the upper threshold [55], i.e., errant NDVI values were assumed to be biased low. The beginning and end of each annual growing season was defined using the pre-defined threshold method (start: when NDVI values first reached 50% of the annual maximum, end: when NDVI values then fell to 50% of the annual maximum), and restricted to be from March to October in each year [56]. NDVI values in forested areas are thought to be contaminated by the signal from the canopy, therefore we used time-integrated NDVI (or the cumulative sum of the differences between NDVI values and the value at the start of the growing season) to represent the net primary production during the growing season [57,58]. Similar to precipitation, we then calculated time-integrated values for two periods: from the start of the growing season through June, and from July to the end of the growing season. Finally, we took the mean values of each metric for all of the pixels in each summer range in each hunting district to represent primary production in a hunting district in a given year. Snow-water equivalent (swe) is a metric of snowpack density on winter ranges for ungulates, and we used swe values estimated from the Snow Data Assimilation System [59]. We calculated the cumulative daily swe values for each pixel on winter range in each hunting district from December 1 to April 31 of each year, then used the mean value for the winter range as an index of winter severity in each hunting district[40,60].

Information on carnivores was available from harvest records (mountain lion and black bears) and annual surveys (wolves). State regulations require that all harvested mountain lions (*Puma concolor*) and black bears (*Ursus americanus*) be presented to FWP staff, and these harvest data were available through all years and for all districts in our study. End-of-the-year minimum wolf (*Canis lupus*) counts (number observed by December 31 of each year) were available as part of the state of Montana’s wolf monitoring program and management plan. We relied on area biologists’ expertise to assign minimum wolf numbers to each elk hunting district. We used the number of mountain lion and black bears harvested and wolf counts directly as a covariate in the models, hypothesizing that they were an index to the underlying populations.

We aggregated data on the fall elk harvest (calves, adult females and adult males) as estimated by the state wildlife agency using annual random telephone surveys of deer and elk license holders. For the analysis of age ratio data, the number of harvested adult females was included as a covariate to attempt to account for the effect of harvest on the age ratio (e.g., high harvest reducing the denominator). For the population model, we included the number of calves, adult females and adult males harvested directly, rather than estimating a relationship between harvest and the age ratio.

All covariates were standardized by centering with the mean and dividing by one standard deviation (S1 Table, see S2 Fig unscaled covariates). Collinearity of the covariates was assessed, and no pairwise comparison exceeded a collinearity threshold of 0.50. For both modeling approaches we fit a single richly parameterized model that included all covariates and their interactions, which allowed for a series of relationships between metrics for precipitation, NDVI, predator abundance and winter severity (S1 File).

### Bayesian analysis

We estimated the parameters of the population model and the age ratio model using a Bayesian framework, which was required for the expression of the multi-step observation process component of the population model. To complete the model statement, we assigned priors to each parameter. Specific to the population model, we assigned a Beta(1,1) prior to adult survival, *ϕ*_*a*_, and we used a Normal(0,1) prior for the intercept *α* on the logit scale. Initial population sizes for each district in each year were assigned a uniform distribution that was left-truncated at the number of animals harvested in the next year, e.g., number of calves in year 1:

$$N_{1,u}^{c}∼Uniform(h_{2,u}^{c},10,000)$$ (10)

Specific to the age ratio model, the intercept (*α*) was assigned a diffuse normal prior (Normal(0,100)). Common to both models, the random effects of year (*ζ*_*t*,*u*_) were given a mean-zero normal prior:

$$\zeta_{t,u}∼Normal(0,\sigma_{\zeta }^{2})$$ (11)

with the variance term ($\sigma_{\zeta }^{2}$) given a uniform prior (Uniform(0,10)).

We fit a single model for each approach rather than relying on model-selection techniques to select a single model out of a model suite. This single model was a richly-parameterized combination of covariates that required 20 (population model) or 21 covariates (age ratio model). The diversity of covariates included interactions between winter severity and environmental conditions during the growing season, primary production, and predator abundance so as to assess the evidence for a relationship between these covariates and winter severity, e.g., low primary production having a stronger effect in severe winters. Thus, we ran the risk of overfitting models to our data if we used independent priors for each regression coefficient. To express an *a priori* belief in parsimony we used a regularization approach to penalize the estimation of regression coefficients. In the Bayesian framework, regularization has the natural expression as setting a common hierarchical prior for all regression coefficients [61]. In our case, we used a normal distribution for all regression coefficients in each model:

$$B=\left[\begin{array}{c} \beta_{spring\;precip}\\ \beta_{summer\;precip}\\ \beta_{spring\;NDVI}\\ \ldots \end{array}\right]∼Normal(0,\sigma_{\beta }^{2})$$ (12)

with a common variance term for all coefficients, $\sigma_{\beta }^{2}$.

We estimated the approximate marginal posteriors of all model parameters using JAGS 4.3.0 program [62] with the runjags package [63] as an interface to the R programming environment [64]. We used random initial values and ran four chains in parallel for both models. Model convergence was graphically assessed using traceplots. The population model required longer MCMC chains, and we used a total of 100,000 iterations with the first 20,000 discarded from each chain, thinning the result for memory issues to keep every fifth sample, resulting in 16,000 samples per chain, or 64,000 total samples. We used 20,000 iterations with the first 5,000 discarded as burnin from each chain for the age ratio model, or 60,000 total samples.

We summarized the approximate posterior distribution of every estimated quantity using the median, and the highest posterior density interval (HPD) to summarize uncertainty. The HPD finds the shortest interval of values for a given density (e.g., a 90% credible interval (CI) therefore corresponds to the shortest range of values that contains 90% of the samples). The estimated regression coefficients apply to covariates that were standardized using the mean and standard deviation and were on the logit scale for the population model, which made direct interpretation difficult. Therefore, in addition to reporting the mean and 90% CI for each standardized coefficient for which we have strong evidence of a relationship with the underlying vital rates, we also included a predicted relationship using the unscaled version of the covariate.

### Goodness-of-fit

Our biological inference was conditional on how well our two modeling approaches can approximate reality. For both approaches, we used posterior predictive checks to compare how well replicated data sets compared to observed data using a discrepancy measure, D [65]. For the linear modeling approach we used an omnibus sum-of-squared residuals metric, where the residual was calculated as the difference between the expected value and either the replicated or observed value:

$$D=\sum_{i=1}^{N}{\left(y_{i}-E(y_{i}|\theta )\right)}^{2}$$ (13)

where the sum is from *i* = 1 to N total data points, *y*_*i*_ is the observed or replicated value, and *E*(*y*_*i*_|*θ*) is the expected value given the parameter values. The population modeling approach was a more-complicated hierarchical model, and two parts of the observation process were of critical interest: the total count for each district-year and (reflecting population size), and the number of calves that ended up in the classified sample (reflecting recruitment). We were particularly interested in evaluating if the model could replicate this significant variation seen in the count data, i.e. accounting for overdispersion in the counts. To evaluate the fit of our model to the total count, we used the Freeman-Tukey statistic as a discrepancy measure to evaluate deviation of observed or replicated counts from expected values:

$$D=\sum_{i=1}^{N}{\left(\sqrt{y_{i}}-\sqrt{E(y_{i}|\theta )}\right)}^{2}$$ (14)

Our data displayed large variation in the number of calves seen in each hunting district and in each year, therefore we used the variance in the number of calves as our final discrepancy measure and compared how well replicated data could reproduce the observed variance. In all of the above cases, one-sided Bayesian p values were calculated as the proportion of MCMC samples where the discrepancy measure for the replicated data was greater than that for the observed data.

## Results

**Fig 3:**
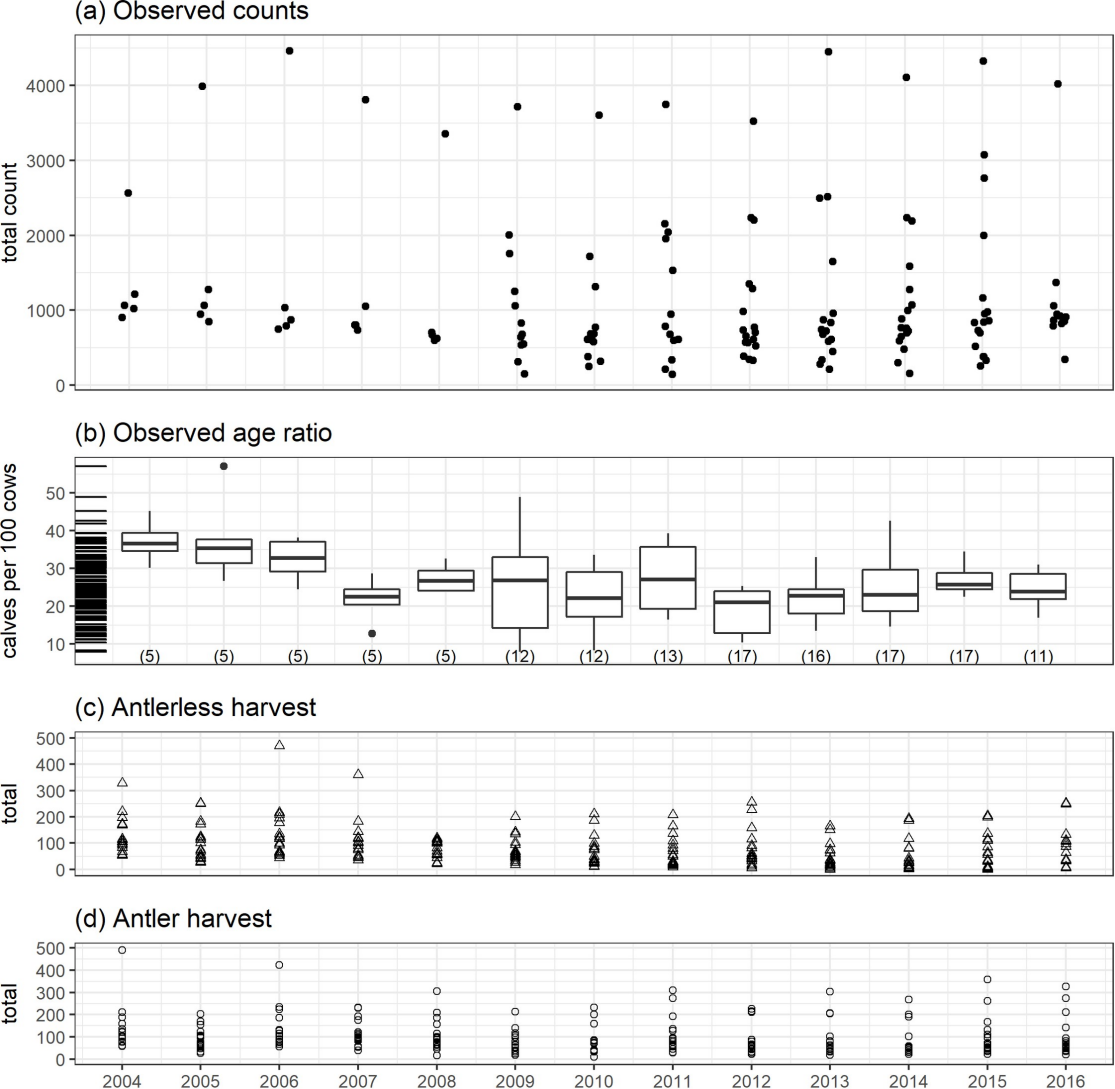
Summary of observed elk count and classification data and estimated antlerless and antlered harvest for the 17 hunting districts included in the elk population model. Both observed counts and age ratios (sample size in parentheses) demonstrated large variation among years and hunting districts. Similarly, antler and antlerless harvest varied through time and district. In panel (a), the observations have been jittered along the x-axis to improve visibility. In panel (b), the y-axis includes a rug that highlights the distribution of the data.

The number of elk counted, observed age ratios, and harvested elk varied considerably among years and hunting districts (Fig 3). Of the 17 hunting districts used for these analyses, counts per hunt district per year ranged annually from a minimum of 147 (HD 214, in 2011) to the maximum of 4,461 (HD 270, in 2006). The average within-hunting-district standard deviation in counts across years was 219.6 (range: 26.9, 513.5). Observed age ratios (mean = 25.3, sd = 8.3, range = 8, 57.1) displayed a similar amount of variation among years with an average within-hunting-district standard deviation of 7.7 (range: 2.4, 11.5). Antlerless and antler harvest varied across years and hunting districts in response to changing regulations over the time period of the study. Notably, high harvest in some districts from 2004 to 2007 was followed by reduced harvest. Our covariate values showed substantial among-year and among-hunting district variation, reflecting a diversity of environmental conditions and indices of predators (S2 Fig).

### Goodness of fit

Our goodness-of-fit metrics did not indicate any obvious lack of fit for the population model or the linear model. The Bayesian p-values for the population model indicated that replicated data sets adequately reproduced the variation observed in both the total counts (p-value = 0.51) and the number of observed calves (p-value = 0.31) (S3 Fig). Similarly, the linear model was an adequate fit to the data (p-value = 0.51) (S4 Fig).

### Sources of variation in recruitment

**Fig 4:**
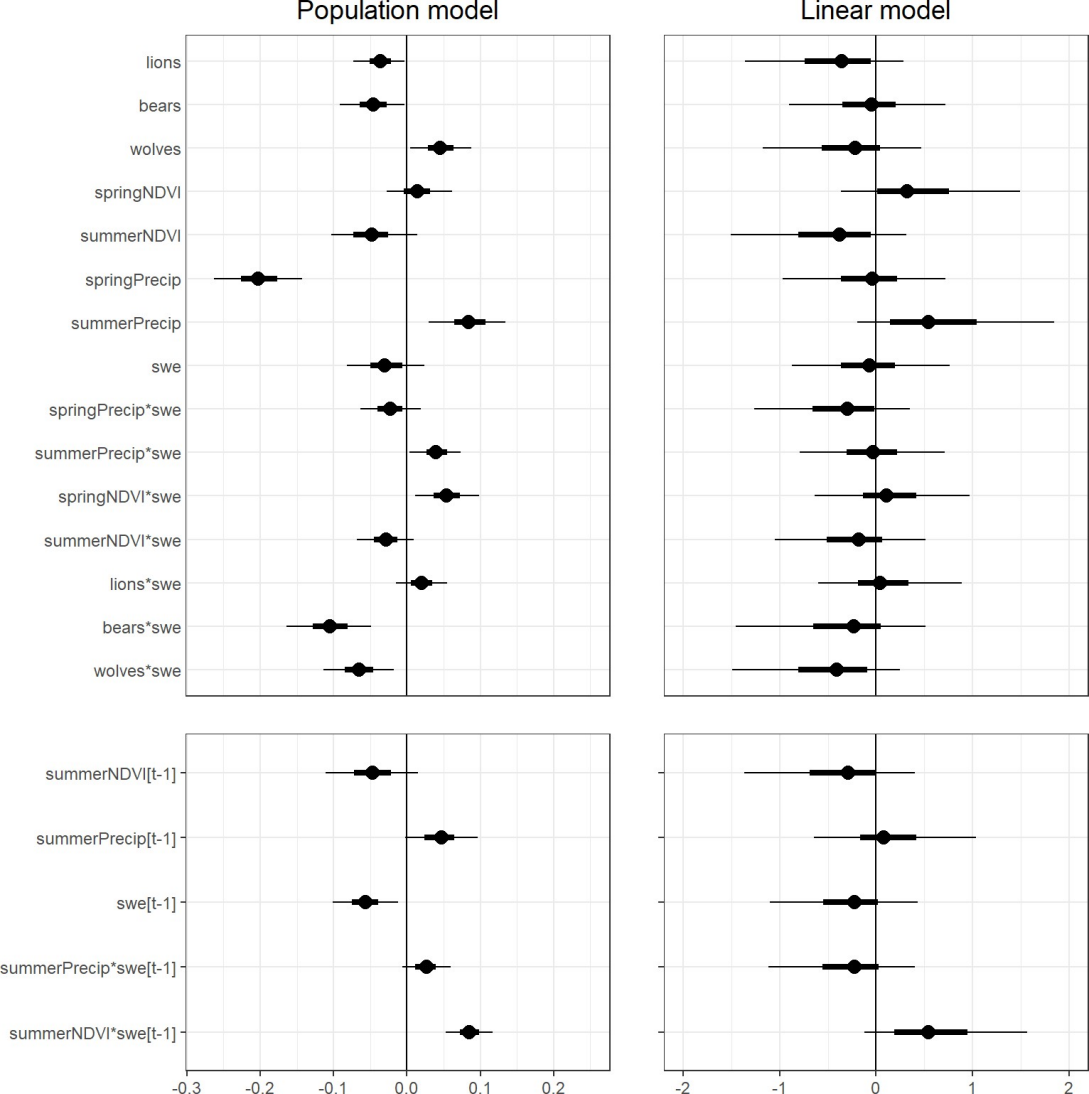
Estimated regression coefficients for standardized covariates. The black dot denotes the median of the approximate posterior distribution, the heavy black line the 50% highest posterior density interval (HPD) and the light black line the 90% HPD interval. The left panel is for the population model, where regression coefficients apply to the per capita recruitment rate and use the logit link. The right panel is for the age ratio model that uses the identity link. The vertical line (0) corresponds to no association with a covariate.

There was a marked difference in the biological inference regarding the effects of covariates on recruitment available from each modeling approach (Fig 4). For the age ratio model, we found very weak evidence for an association between our covariates and recruitment. Though the point estimates of several covariates (e.g., summerPrecip) are suggestive of an underlying relationship, the very broad 50% and 90% highest posterior density intervals all overlap zero, which prevented strong inference in each case.

In contrast, we found very strong evidence for a series of relationships between covariates and recruitment using the population model (S2 Table). For an average year and with all covariates held to their average value (zero for standardized covariates), our model predicted an overall mean recruitment rate of 0.25 (90% CI = [0.21, 0.29], hereafter all similar credible intervals denoted as [low, high]). For each covariate below, we report the estimated effect on the logit scale and then a prediction of how recruitment changed from this overall mean as that covariate increased/decreased one standard deviation from the average value. We found a weak negative association between mountain lion harvest and per capita recruitment rates (${\hat{\beta }}_{lions}=-0.04[-0.07,0]$), which corresponded to a decline in per capita recruitment from the overall mean of 0.25 [0.21, 0.29] at the average lion harvest (4.12 harvested per hunting district) to 0.24 [0.19, 0.27] at one standard deviation above the average lion harvest (7.88 harvested) (S5 Fig). Similarly, we found a weak association between black bear harvest and per capita recruitment rates (${\hat{\beta }}_{bears}=-0.05[-0.09,0]$), declining from the overall mean (0.25 [0.21, 0.29]) at the average black bear harvest (21.31 harvested per hunting district) to 0.24 [0.21, 0.28] at one standard deviation above the average black bear harvest (39.17 harvested). However, we found strong evidence for an interaction with cumulative snow water equivalent (swe) (${\hat{\beta }}_{bears*swe}=-0.11[-0.16,-0.05]$) that became different from zero only at higher bear harvests and more severe winters. At the average black bear harvest, per capita recruitment rates in a mild winter (hereafter defined as the 5th percentile of standardized swe values, swe = -0.95), average winter (swe = 0), or severe winter (hereafter defined by the 95th percentile of swe values, swe = 2.22) showed no meaningful difference. At one standard deviation above the average black bear harvest recruitment in a mild winter was higher than in a mean winter (difference = 0.02 [0.01, 0.04]), and even higher than in a severe winter (difference = 0.07 [0.03, 0.12]) (S6 Fig). In contrast, we found a weak positive association between wolf counts and recruitment (${\hat{\beta }}_{wolves}=0.05[0,0.09]$), increasing from the overall mean (0.25 [0.21, 0.29]) at the average wolf count (15.99 wolves) to 0.26 [0.22, 0.30] at one standard deviation above the average wolf count (30.49 wolves). However, we also found strong evidence for a negative interaction with cumulative snow water equivalent (${\hat{\beta }}_{wolves*swe}=-0.06[-0.11,-0.02]$) such that recruitment declined with high wolf counts at a faster rate as winter severity increased increasing winter severity. At one standard deviation above the average wolf count recruitment in a mild winter was higher than in a mean winter (difference = 0.02 [0.01, 0.03]), and even higher than in a severe winter (difference = 0.06 [0.03, 0.09]) (S7 Fig).

We also found strong evidence for an association between several environmental covariates that corresponded to conditions during the first year the calf is on the ground and per capita recruitment. Cumulative spring precipitation had a negative association with per capita recruitment rates (${\hat{\beta }}_{springPrecip}=-0.2[-0.26,-0.14]$), declining from the overall mean at the average spring precipitation (0.17 m) to 0.21 [0.18, 0.25] at one standard deviation above the average spring precipitation (0.22 m) (S8 Fig). In comparison, cumulative summer precipitation had a weaker positive association with recruitment (${\hat{\beta }}_{summerPrecip}=0.08[0.03,0.13]$), increasing from the overall mean at the average summer precipitation (0.15 m) to 0.27 [0.23, 0.31] at one standard deviation above the average summer precipitation (0.19 m), and strong evidence for an interaction with winter severity (${\hat{\beta }}_{summerPrecip*swe}=0.04[0,0.07]$) such that low values of summer precipitation combined with winter severity to reduce per capita recruitment. At one standard deviation below the average summer precipitation (0.11 m), recruitment was higher in a mild winter than in an average one (difference = 0.02, [0.01, 0.03]), and even higher than in a severe winter (difference = 0.04, [0.01, 0.07]) (S9 Fig). Although we found no evidence for a main effect of spring NDVI, we found evidence for an interaction with winter severity (${\hat{\beta }}_{springNDVI*swe}=0.05[0.01,0.1]$). Low values of spring NDVI combined with severe winters were associated with reduced recruitment. At one standard deviation below the average spring NDVI (0.81), recruitment was again higher in a mild winter than an average winter (difference = 0.02 [0, 0.03]) and a severe winter (difference = 0.05 [0.01, 0.08]) (S10 Fig).

Finally, we also found strong evidence for an association between environmental variation during the year in which the calf is in-utero and recruitment. We found strong evidence for a negative association with lagged winter severity and per capita recruitment rates (${\hat{\beta }}_{swe[t-1]}=-0.06[-0.1,-0.01]$), declining from the overall mean at the average swe (8.15 m) to 0.23 [0.20, 0.28] at one standard deviation above the average swe (14.45 m). Although we did not find evidence for a main effect of summer NDVI, we found strong evidence for an interaction with winter severity (${\hat{\beta }}_{summerNDVI*swe[t-1]}=0.08[0.05,0.12]$) such that recruitment at low summer NDVI (1 standard deviation below the mean) was higher in a mild winter than a mean winter (difference = 0.03 [0.02, 0.04]), and considerably higher than in a severe winter (difference = 0.08 [0.05, 0.12]) (S11 Fig).

### Evidence for variation in vital rates: Differences between the two approaches

**Fig 5:**
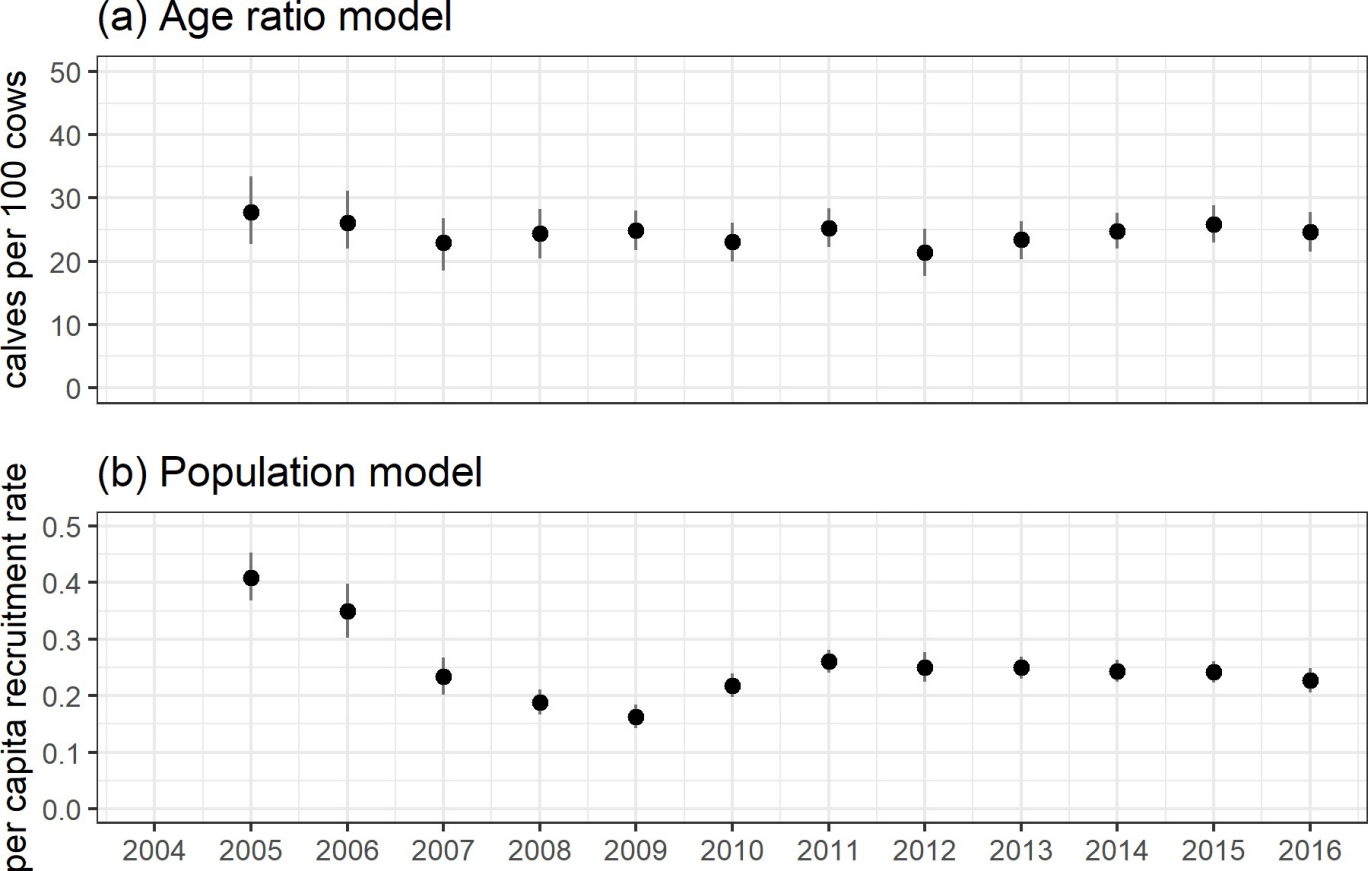
**Estimated average values among years for (a) age ratios, and (b) per capita recruitment rates.** These values were calculated by generating an approximate posterior distribution for the average value (age ratio or rate) across hunting districts within a year. The black dot denotes the median of the approximate posterior distribution, the heavy black line the 50% highest posterior density interval (HPD) and the light black line the 90% HPD interval.

**Fig 6:**
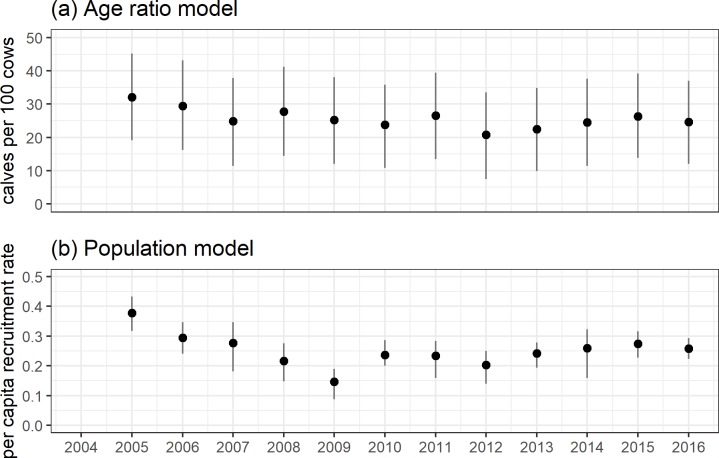
**Estimated yearly random effects for (a) age ratios, and (b) per capita recruitment rates.** These values were calculated by holding all other covariates to their mean (zero) and represent predicted values for average conditions. The black dot denotes the median of the approximate posterior distribution, the heavy black line the 50% highest posterior density interval (HPD) and the light black line the 90% HPD interval.

In contrast to the population model, the variance term for the age ratio model incorporated process variance and observation error ($\sigma_{age\;ratio}^{2}=7.19[6.4,8.03]$), and translated into large uncertainty in estimated average age ratios (Fig 5). In contrast, the population model had a separate error term to account for variation in the observation process conditional on the underlying population size ($\sigma_{count}^{2}=6.7[6.67,6.78]$), and the partitioning of these errors significantly reduced undcertainty in the estimated underlying average yearly per capita recruitment rate (Fig 5). Similarly, we found very weak evidence to support a random effect of year for the age ratio model ($\sigma_{\zeta }^{2}=2.52[0.17,4.49]$) and strong evidence for among-year variation otherwise unaccounted for by covariates using the population model ($\sigma_{\zeta }^{2}=0.37[0.23,0.55]$). With all other covariate values held to their mean the predicted values from the age ratio model highlight the lack of evidence for a yearly effect in ratios. However, the population model strongly suggested a decline in per capita recruitment from 2004–2009, followed by stability (2010–2016) (Fig 6).

### Understanding population dynamics

**Fig 7:**
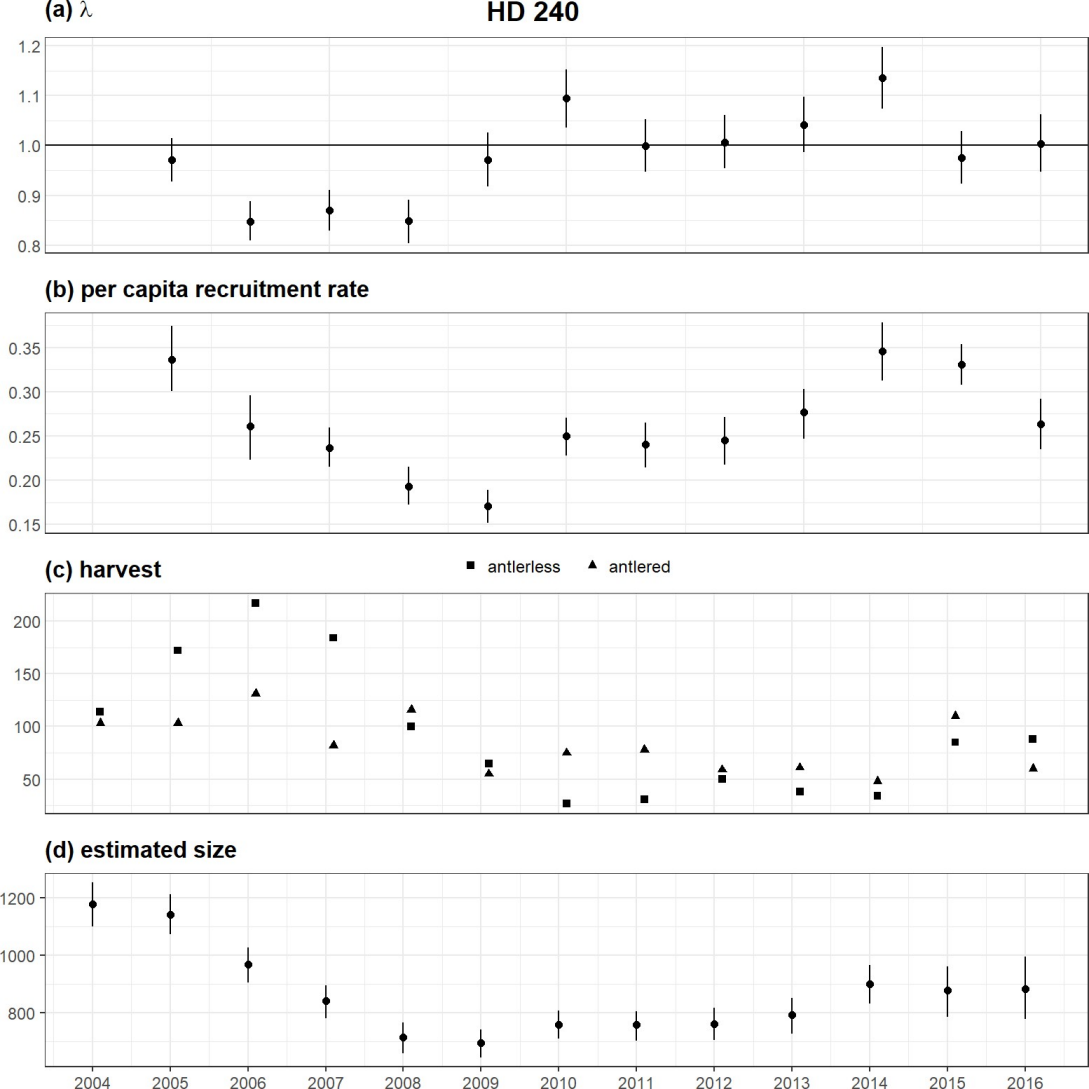
Demographic summary of an example hunting district (HD 240), including population growth rate, per capita recruitment rate, observed harvest, and predicted/observed size. The black dot denotes the median of the approximate posterior distribution, the heavy black line the 50% highest posterior density interval (HPD) and the light black line the 90% HPD interval.

In addition to an improved understanding of the sources of variation in vital rates (Figs 4, 5 and 6), the population model also allowed deeper insights into population dynamics. By linking the numbers in each age/sex class through time via biological processes (i.e., survival and reproduction), derived quantities can be calculated that are of direct interest for wildlife management (Fig 7). For example, the estimated sum of all age/sex classes, the total *N*_total_, provided a qualitative ability to assess the quality of the observation process. If observed counts are markedly different that the predicted number of animals in a population, it indicates a lack of model fit to the observation process, which can arise from a diversity of factors, including double-counting (in the case of an overestimate), or partial counting (in the case of an underestimate). It can also indicate a lack of fit due to a violation of the closure assumption from immigration/emigration, which can inform how populations are defined in the management process. Moreover, estimated sizes ($N_{t}^{total}$) through time also provide insight into population growth rates, $\lambda_{t}=\frac{N_{t}^{total}}{N_{t-1}^{total}}$, provided they are a consistent index of the true, unknown population size (Fig 7). Where populations are managed using harvest as the primary tool, a comparison of harvest numbers to estimated values of *λ* through time provides insight into the efficacy of harvest regulations on management objectives indexed by population growth rates.

## Discussion

Our results demonstrate how using a population model to treat monitoring data as a time-series of observations connected by biological processes can improve biological inference into sources of variation in vital rates, as well as provide information on population dynamics, resulting in useful information to aid management. A population model developed from routinely collected elk count, classification and harvest data provides information regarding not only recruitment, but also, estimates of population growth rate. The population modeling approach provided insights regarding factors affecting recruitment that may also inform management decisions. We found that per capita recruitment rates are most strongly associated with spring and summer precipitation, and to a lesser extent associated with indices of winter severity, predator populations, and primary production.

Our results strongly support prior conclusions that using a population model to analyze time series of data yields higher statistical power and richer insight into biological processes compared to a linear model [27,30]. We attribute the improvement to the separation of observation error from variance in the underlying biological processes, as well as using demographic rates to connect observations through time. Although prior work has suggested that ignoring observation error can have minimal impact on evaluating the strength of support for covariates, observation error is likely to have an important impact where it is large or varies among data points [30]. This is particularly relevant for managers, given that long-term monitoring data are often the result of varying degrees of effort and survey conditions, different survey protocols, resulting in variability in the observation process and associated sightability. Biological inference from this approach is thought to be robust to deviations from the true demographic model [27,30]. This is a critical observation for managers, as the integration of data from different sources can require pooling of some age/sex classes in the model. For example, the count of adult females in our work in the late spring is contaminated by the presence of yearling females that have a lower and much more variable probability of conception [66].

As a result of higher statistical power, the population model provided insights into factors affecting recruitment that were not detected using the age ratio approach. We found that environmental conditions experienced by the calf on the ground (year t, related to calf survival) and the female prior to conception and when the calf is in-utero (year t-1) were strongly connected to per capita recruitment rates. Contrary to our expectations, cumulative spring precipitation in year t was negatively associated with recruitment. A post-hoc analysis of the precipitation signal strongly suggested that these high values of spring precipitation were the result of heavy snow on the summer range, an observation consistent with previous work on elk in this system [7]. Cold and wet springs are thought to be a risk factor for elevated neonatal mortality, as environmental conditions interact to predispose neonates to illness, delay green-up and increase risk of predation [67,68]. Summer precipitation during year t and year t -1 was strongly, positively associated with recruitment. We also found evidence to support an interaction between summer precipitation values and winter severity in year t such that dry summers interacted with particularly severe winters to diminish calf survival in year t. Precipitation is known to be related to the rate of forage senescence, digestible energy and relative protein content [69–71], key factors in determining the body condition of ungulates headed into winter [38,72,73]. Our results are consistent with previous work concluding that fall body condition affects pregnancy rates, overwinter calf survival, and neonate survival in the following year [38]. In contrast to previous work that found the relationship between precipitation and recruitment to be relatively minor [7], we found spring and summer precipitation to be major contributors to variation in recruitment supporting the importance of summer forage conditions to calf production, maternal provisioning, and survival. We also attribute our ability to detect these relationships to our separation of precipitation into the two phases of spring (an index of early growing/environmental conditions) and summer (as an index of forage quality headed into winter). The use of a season-long precipitation metric could conflate variation in these two periods such that only the most extreme combination would be associated with variation in recruitment.

We found mixed evidence for a relationship between primary production (NDVI) and per capita recruitment rates. Although we found no evidence for a direct relationship between NDVI in either in the spring or summer during the year the calf is on the ground and recruitment, we did find evidence for an interaction between spring NDVI and winter severity such that years with combined low spring NDVI and severe winters were associated with lower recruitment. Moreover, we found an interaction between summer NDVI and winter severity during the year the calf is in-utero (year t-1) that suggested that high values of summer NDVI and severe winters reduced recruitment. NDVI is frequently interpreted as an index of forage quality [53], though the link between the two is uncertain and can depend on the NDVI metric used [74–76]. Spring green-up as indexed by increasing NDVI values has been positively associated with body condition [77], as the greening vegetation has high digestible energy and protein content, and the relative value of this phase of forage quality has been suggested as a driver of spring migrations [78]. We used a time-integrated NDVI metric where low values likely corresponded to a delayed start of season and found they only become meaningful when followed by a severe winter, consistent with other work highlighting the interactive effects of nutrition and winter severity [40,79], and broadly suggesting that calves can otherwise make up for a poor start in mild winter conditions. We also found strong evidence that summer NDVI and winter severity in year t-1 were related to recruitment through an interaction such that high values of summer NDVI in a severe winter were negatively associated with recruitment. This is not the first study to document a surprising relationship between NDVI and the demographic performance of ungulates [7], and highlights the care that must be taken in assuming NDVI represents the same biological process across a growing season. The relationship between NDVI and forage quality may be fundamentally different in late summer when the high NDVI corresponds to diminished digestible energy [75]. Alternatively, we speculate that summer NDVI values might be correlated to large scale, long-term weather patterns such that they are serving as a proxy for environmental conditions in the approaching winter. Further work is required to detail the link between NDVI and forage quality as it relates to ungulate nutrition and body condition, and we caution against the assumption that high NDVI values area a proxy for high-quality ungulate forage.

In addition to the important influences of environmental conditions on calf recruitment, predation has been shown to be a major factor influencing juvenile elk survival in individual-based studies that allow for the estimation of cause-specific mortality [10,44]. It is considerably more challenging to assess the effects of predators on vital rates when working at the population level, given accurate predator population estimates are difficult to attain and the effects of predation can be complicated by interacting effects with weather and resource limitation. In particular, studies need to be carefully designed when trying to assess how the harvest of predators is related to variation in the vital rates of prey [80]. The connection between predator harvest, predator population dynamics and predation risk to ungulates is unclear and has rarely been evaluated [81]. This lack of clarity is worsened where predator harvest regulations are set in response to a combination of social, biological, and political factors or where harvest can fluctuate in response to any factors unrelated to population vital rates. In such cases, harvest numbers are a poor reflection of underlying predator population dynamics [82–84]. Given this uncertainty, results from models that include predator harvest as covariates should be interpreted with care as the relationship may be spurious. Furthermore, they should not be interpreted as indicative of predation pressure, merely suggestive of a potential relationship that requires further investigation. With that in mind, the negative relationship between black bear harvest and recruitment found here is consistent with harvest numbers being an index to population size. For black bears, predation is thought to occur primarily during the neonate phase in late spring/early summer [44], and high harvest the following fall and spring may serve as a reasonable proxy for the population size of black bears during the birth pulse, although we stress that further work is needed to clarify the relationship between harvest and predation pressure. On the other hand, we found a weak positive association between minimum wolf counts, a more direct index of population size, and recruitment that we interpret as a spatial arrangement of predators on the landscape to take advantage of more productive areas [85]. That signal was swamped, however, by the interaction between wolf counts and winter severity that suggested high wolf counts interacted with severe winters to reduce recruitment. This result is consistent with prior work in the region [3] (but see [86]), and we speculate that it may reflect an additive effect of predation to nutritional and environmental stress during severe winters, when elk likely become more vulnerable to wolf predation. We stress that more work is needed to understand the relationship between minimum wolf counts, wolf abundance and vital rates. More generally, we echo the caution that adequately understanding the connections between predator indices (harvest or counts), predator population dynamics and ungulate vital rates requires carefully designed experiments [80].

Using derived recruitment estimates from population models, rather than estimated age ratios, is a practical alternative for managers and uses routinely collected survey data. However, the results are subject to a series of potential biases and need to be carefully interpreted. It is unknown how the detection process is related to the actual (latent) abundance of elk. If a fraction of the population is persistently unavailable during surveys estimates of the population size from a count-based model are biased low. Yet, provided that the observed population is a consistent index of the size of the total population, estimated population growth rates and trends and underlying vital rates should be unbiased [35]. Count data are also subject to mis-classification errors of juvenile and adult females that can bias resulting estimates of vital rates. More work is needed to evaluate the consequences of the mis-specification of the underlying biological processes and the parameterization of the observation process to understanding population dynamics [31,35]

### Management implications

We recommend managers consider using routinely collected time-series of observed count data and harvest data in a population model. This approach, as compared to the age ratio modeling approach or monitoring of trends in observed count and age ratios over time provides improved biological inference into sources of variation in vital rates, as well as critical information on population dynamics, resulting in useful information to aid management decisions. The population model approach allows managers to estimate population growth rates and use model predictions and goodness-of-fit metrics to inform the survey methodology. Furthermore, we suggest that any lack of fit between the model and the observations can be highly informative for managers. Poor fit can indicate where the closure assumptions are violated due to emigration or immigration and challenge ideas about the delineation of populations by informing managers about the quality of the observation process. This framework is also flexible enough to accommodate data that are missing due to either logistical limitations that prevented a yearly survey or to an age/class structure that is partly unobservable, such that managers can estimate temporal trends in populations with discontinuous or incomplete data. Combined, the practical benefits of the population model approach render it an attractive option for the informed management of populations using routinely collected survey data.

## Supporting information

S1 Fig Hunting districts used in the analysis. We restricted our analysis to those hunting districts with at least 6 years of data over the duration of the study (2004 to 2016). The resulting subset of elk hunting districts used for analysis is depicted as the shaded gray hunting districts.
(TIF)

S2 Fig Unscaled covariate values as a function of time. (TIF)

S3 Fig Goodness-of-fit metrics for the population model. We used posterior predictive checks: 1) to compare variation in the observed total counts of animals to replicated total counts using the Freeman-Tukey statistic as a discrepancy measure (left panel), and, 2) to compare variance in the observed number of calves to the variance in the observed number of calves (right panel, red line indicates the observed value).
(TIF)

S4 Fig Goodness-of-fit metrics for the linear model. We used a posterior predictive check to compare variation in the observed age ratios (calves:100 adult females) to replicated age ratios using the sum-of-squared-residuals as a discrepancy measure.
(TIF)

S5 Fig Predicted relationship between per capita recruitment rates and mountain lion harvest. Mountain lion harvest was on a standardized scale, with 0 corresponding to the average mountain lion harvest (4.0 harvested) and 1 corresponding to one standard deviation of harvest above the average (8.9 harvested) (top panel). The second panel shows the predicted difference in recruitment rates between a mild winter (swe = 5th percentile of observed values) and a mean winter (swe = 0) as a function of mountain lion harvest, and the bottom panel shows the difference in recruitment rates between a mild winter and a severe winter (swe = 95th percentile of observed values).
(TIF)

S6 Fig Predicted relationship between per capita recruitment rates and black bear harvest. Black bear harvest was on a standardized scale, with 0 corresponding to the average black bear harvest (20.7 harvested) and 1 corresponding to one standard deviation of harvest above the average (38 harvested) (top panel). The second panel shows the predicted difference in recruitment rates between a mild winter (swe = 5th percentile of observed values) and a mean winter (swe = 0) as a function of black bear harvest, and the bottom panel shows the difference in recruitment rates between a mild winter and a severe winter (swe = 95th percentile of observed values).
(TIF)

S7 Fig Predicted relationship between per capita recruitment rates and wolf counts. Wolf counts were on a standardized scale, with 0 corresponding to the average (15.8 wolves) and 1 corresponding to one standard deviation above the average (30.4 wolves) (top panel). The second panel shows the predicted difference in recruitment rates between a mild winter (swe = 5th percentile of observed values) and a mean winter (swe = 0) as a function of wolf numbers, and the bottom panel shows the difference in recruitment rates between a mild winter and a severe winter (swe = 95th percentile of observed values).
(TIF)

S8 Fig Predicted relationship between per capita recruitment rates and cumulative spring precipitation on summer range. Spring precipitation was on a standardized scale, with 0 corresponding to the average (0.18 m) and 1 corresponding to one standard deviation above the average (0.22 m) (top panel). The second panel shows the predicted difference in recruitment rates between a mild winter (swe = 5th percentile of observed values) and a mean winter (swe = 0) as a function of spring precipitation, and the bottom panel shows the difference in recruitment rates between a mild winter and a severe winter (swe = 95th percentile of observed values).
(TIF)

S9 Fig Predicted relationship between per capita recruitment rates and cumulative summer precipitation on summer range. Summer precipitation was on a standardized scale, with 0 corresponding to the average (0.15 m) and 1 corresponding to one standard deviation above the average (0.19 m) (top panel). The second panel shows the predicted difference in recruitment rates between a mild winter (swe = 5th percentile of observed values) and a mean winter (swe = 0) as a function of summer precipitation, and the bottom panel shows the difference in recruitment rates between a mild winter and a severe winter (swe = 95th percentile of observed values).
(TIF)

S10 Fig Predicted relationship between per capita recruitment rates and spring time-integrated NDVI on summer range. Spring NDVI was on a standardized scale, with 0 corresponding to the average (1.06) and 1 corresponding to one standard deviation above the average (1.40) (top panel). The second panel shows the predicted difference in recruitment rates between a mild winter (swe = 5th percentile of observed values) and a mean winter (swe = 0) as a function of spring NDVI, and the bottom panel shows the difference in recruitment rates between a mild winter and a severe winter (swe = 95th percentile of observed values).
(TIF)

S11 Fig Predicted relationship between per capita recruitment rates and summer time-integrated NDVI on summer range, lagged one year. Summer NDVI was on a standardized scale, with 0 corresponding to the average (4.10) and 1 corresponding to one standard deviation above the average (4.85) (top panel). The second panel shows the predicted difference in recruitment rates between a mild winter (swe = 5th percentile of observed values) and a mean winter (swe = 0) as a function of lagged summer NDVI, and the bottom panel shows the difference in recruitment rates between a mild winter and a severe winter (swe = 95th percentile of observed values).
(TIF)

S1 Table Summary statistics for covariates across all hunting districts and years. (DOCX)

S2 Table Summaries of the approximate posterior distributions for regression coefficients. (DOCX)

S1 File Detailed model statement. (DOCX)
