# Supplementary figures and images for: An improved understanding of ungulate population dynamics using count data: Insights from western Montana
Source: PLoS One. 2019 Dec 23;14(12):e0226492. doi: 10.1371/journal.pone.0226492 (PMC6927647; doi:10.1371/journal.pone.0226492)

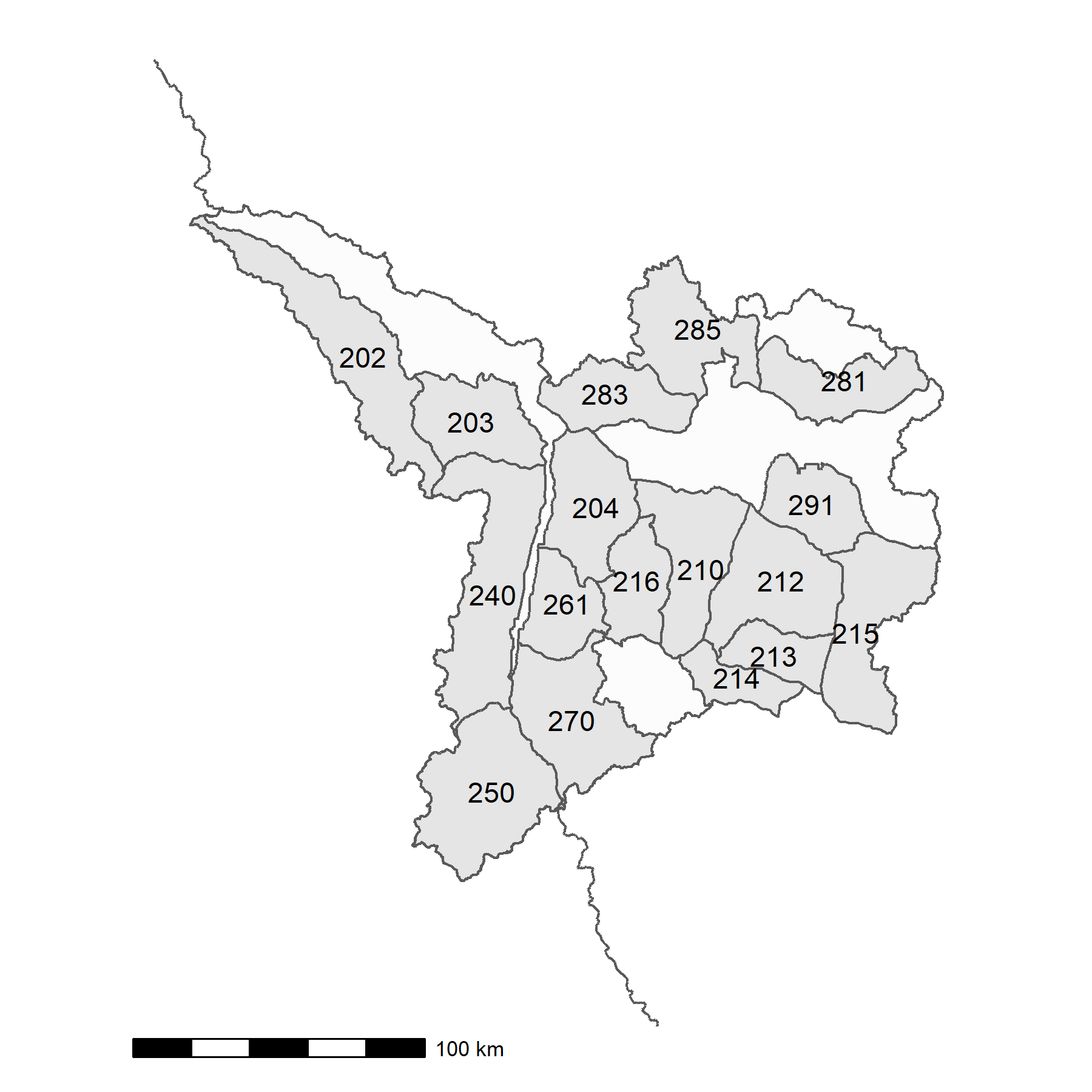

Supplement: S1 Fig — We restricted our analysis to those hunting districts with at least 6 years of data over the duration of the study (2004 to 2016). The resulting subset of elk hunting districts used for analysis is depicted as the shaded gray hunting districts. (TIF) [file pone.0226492.s001.tif]

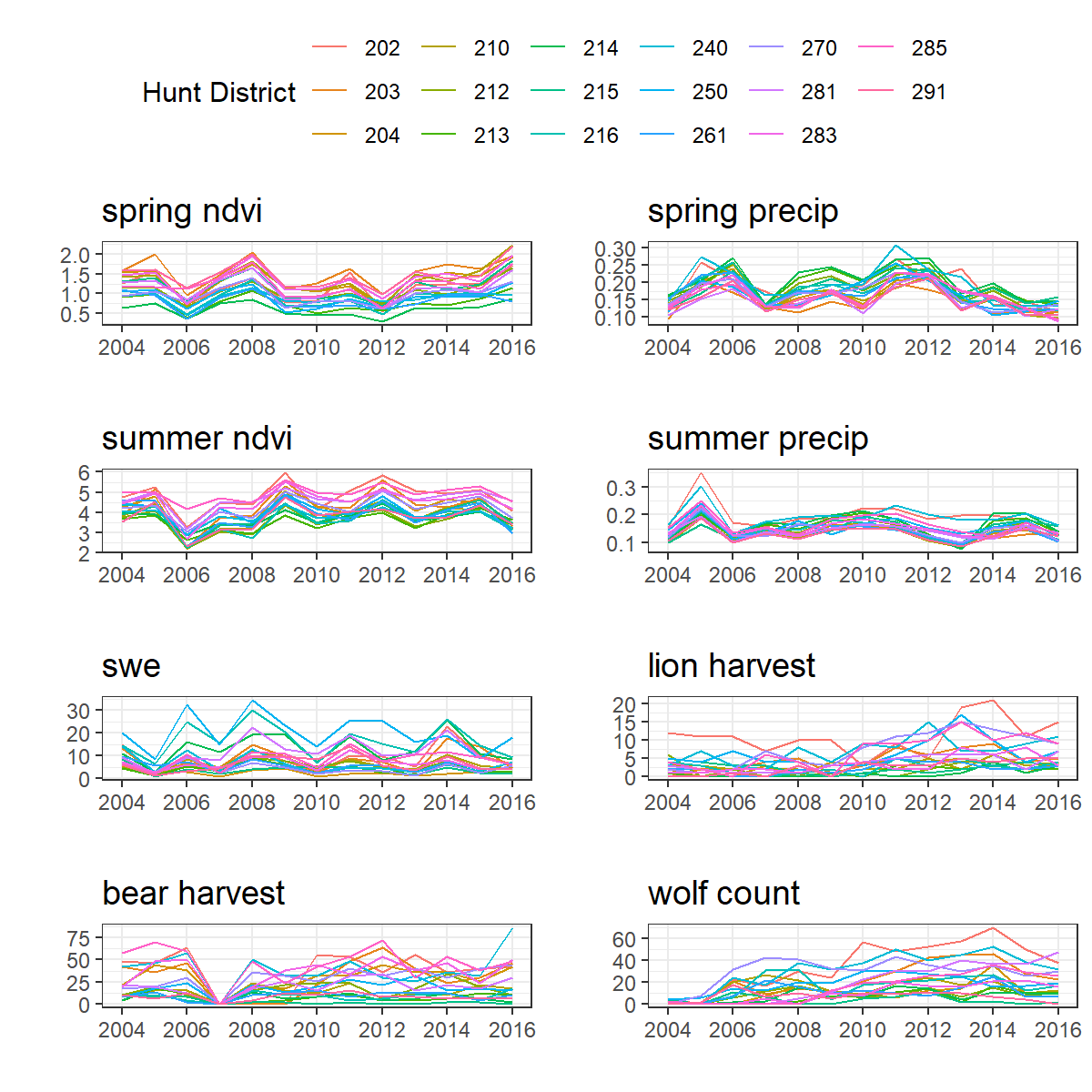

Supplement: S2 Fig — (TIF) [file pone.0226492.s002.tif]

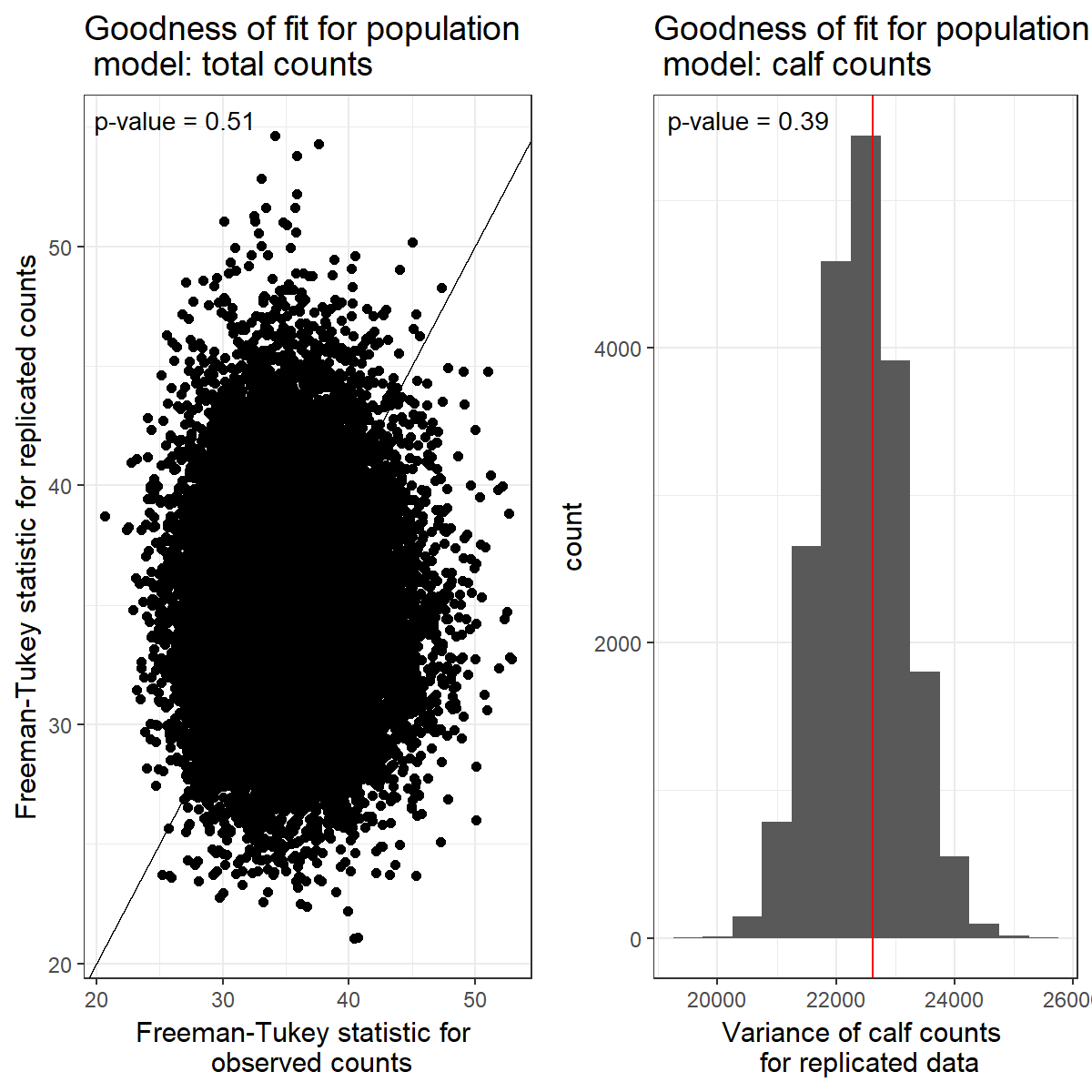

Supplement: S3 Fig — We used posterior predictive checks: 1) to compare variation in the observed total counts of animals to replicated total counts using the Freeman-Tukey statistic as a discrepancy measure (left panel), and, 2) to compare variance in the observed number of calves to the variance in the observed number of calves (right panel, red line indicates the observed value). (TIF) [file pone.0226492.s003.tif]

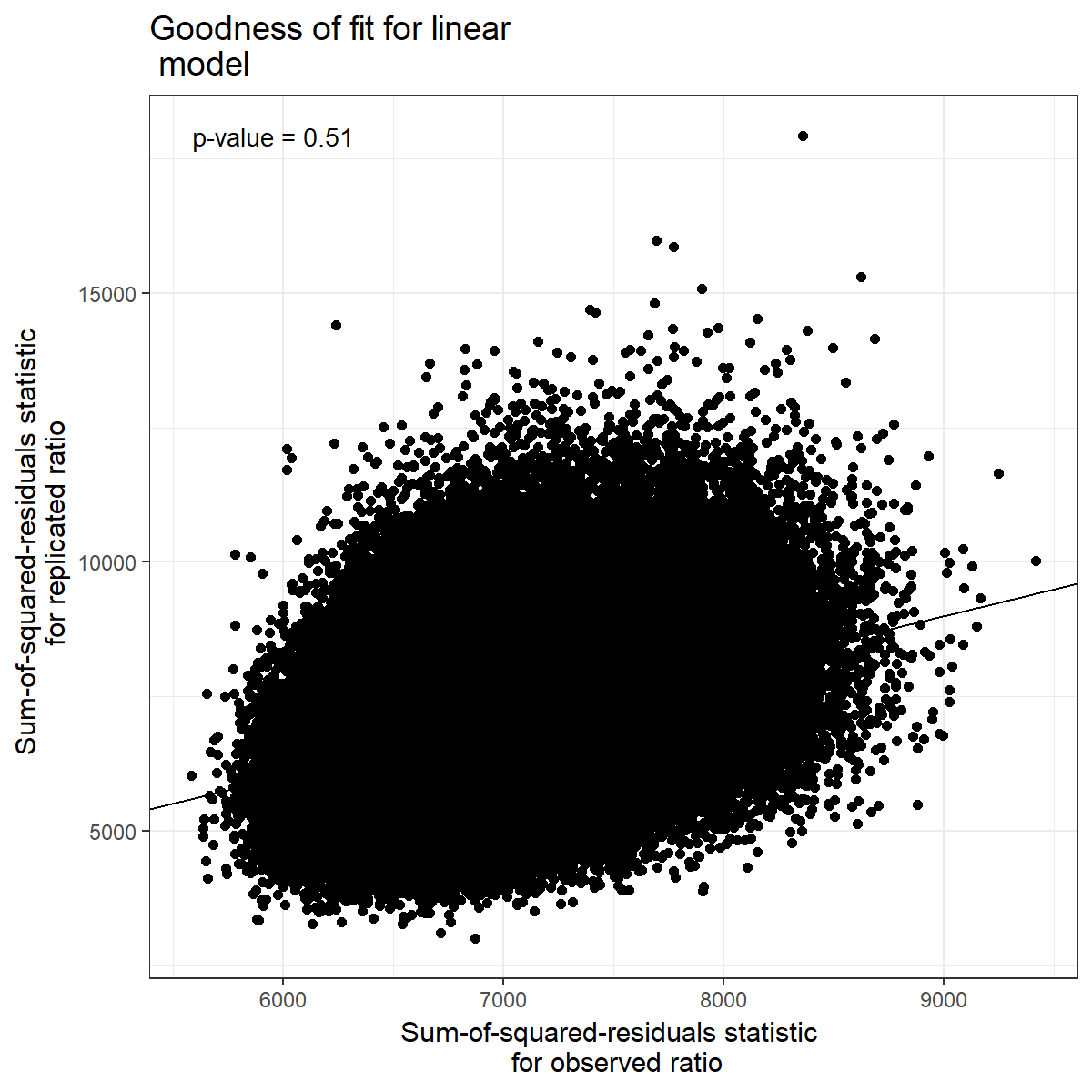

Supplement: S4 Fig — We used a posterior predictive check to compare variation in the observed age ratios (calves:100 adult females) to replicated age ratios using the sum-of-squared-residuals as a discrepancy measure. (TIF) [file pone.0226492.s004.tif]

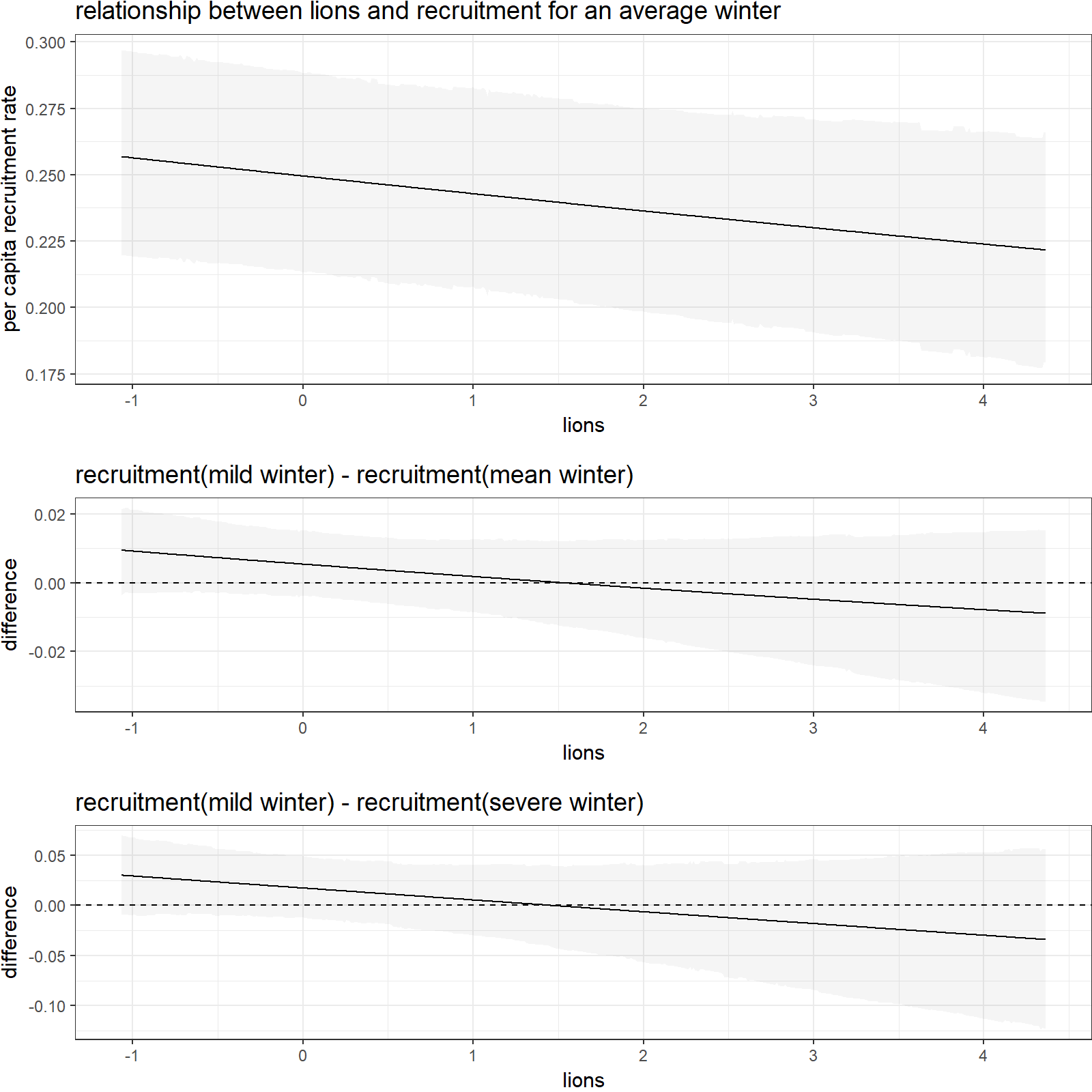

Supplement: S5 Fig — Mountain lion harvest was on a standardized scale, with 0 corresponding to the average mountain lion harvest (4.0 harvested) and 1 corresponding to one standard deviation of harvest above the average (8.9 harvested) (top panel). The second panel shows the predicted difference in recruitment rates between a mild winter (swe = 5th percentile of observed values) and a mean winter (swe = 0) as a function of mountain lion harvest, and the bottom panel shows the difference in recruitment rates between a mild winter and a severe winter (swe = 95th percentile of observed values). (TIF) [file pone.0226492.s005.tif]

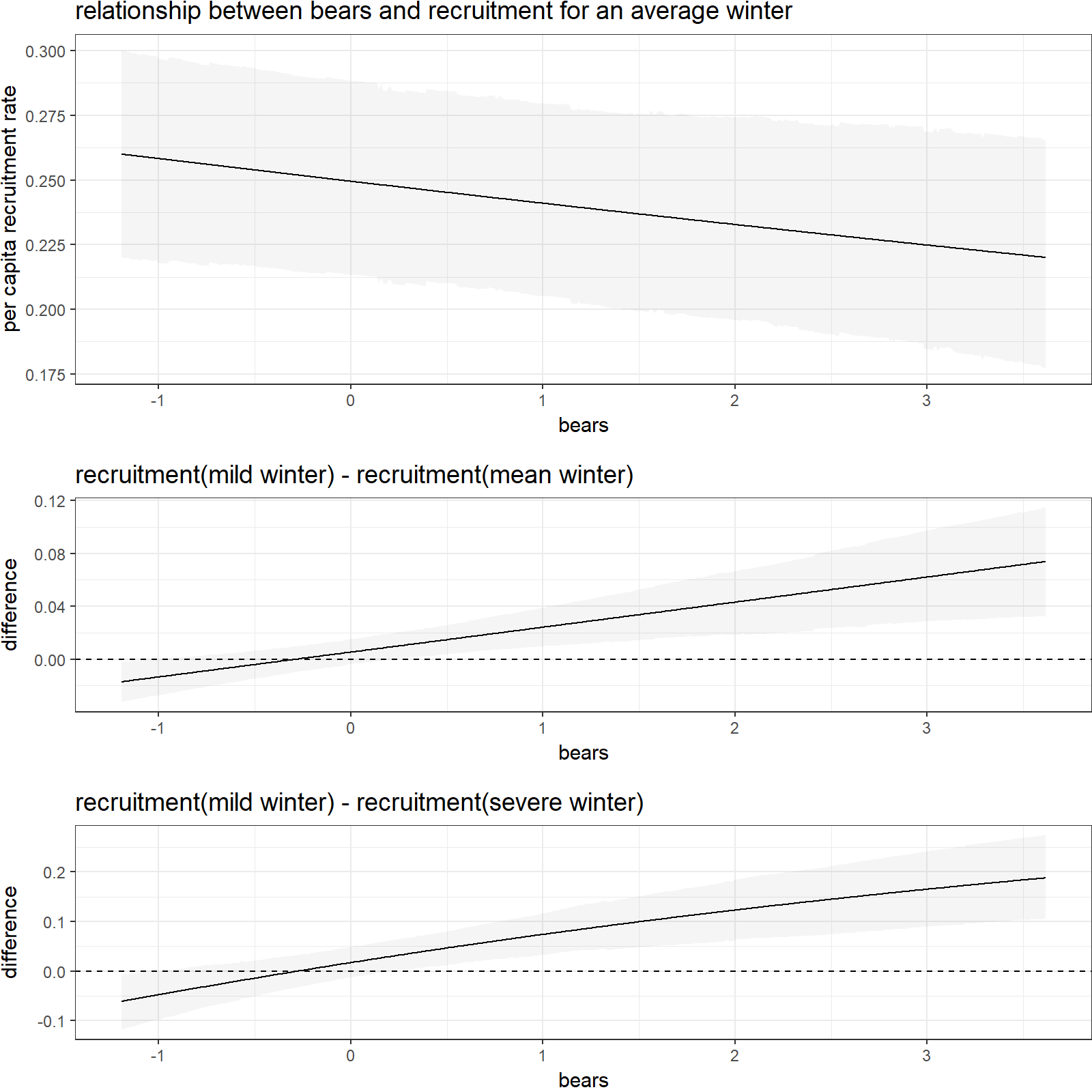

Supplement: S6 Fig — Black bear harvest was on a standardized scale, with 0 corresponding to the average black bear harvest (20.7 harvested) and 1 corresponding to one standard deviation of harvest above the average (38 harvested) (top panel). The second panel shows the predicted difference in recruitment rates between a mild winter (swe = 5th percentile of observed values) and a mean winter (swe = 0) as a function of black bear harvest, and the bottom panel shows the difference in recruitment rates between a mild winter and a severe winter (swe = 95th percentile of observed values). (TIF) [file pone.0226492.s006.tif]

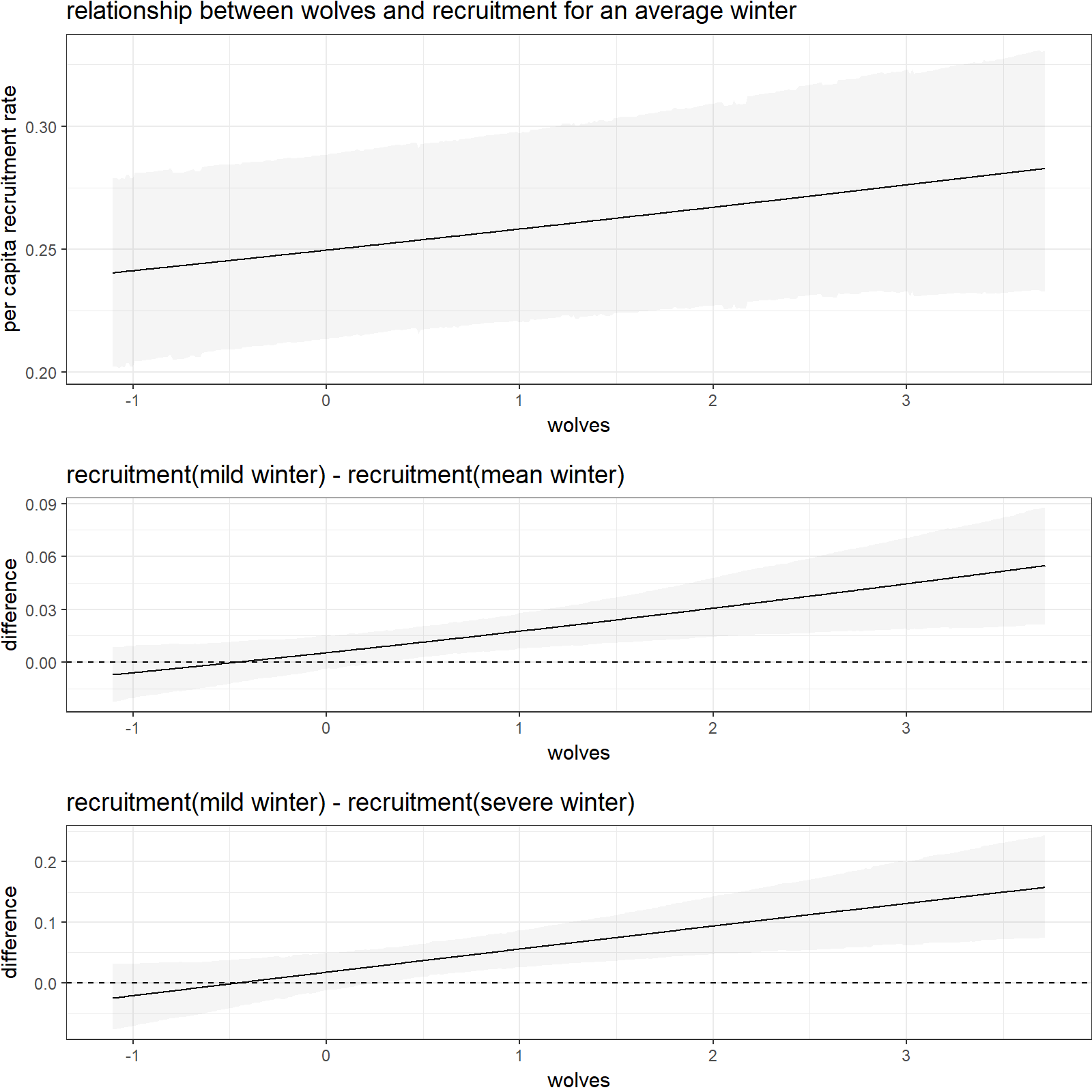

Supplement: S7 Fig — Wolf counts were on a standardized scale, with 0 corresponding to the average (15.8 wolves) and 1 corresponding to one standard deviation above the average (30.4 wolves) (top panel). The second panel shows the predicted difference in recruitment rates between a mild winter (swe = 5th percentile of observed values) and a mean winter (swe = 0) as a function of wolf numbers, and the bottom panel shows the difference in recruitment rates between a mild winter and a severe winter (swe = 95th percentile of observed values). (TIF) [file pone.0226492.s007.tif]

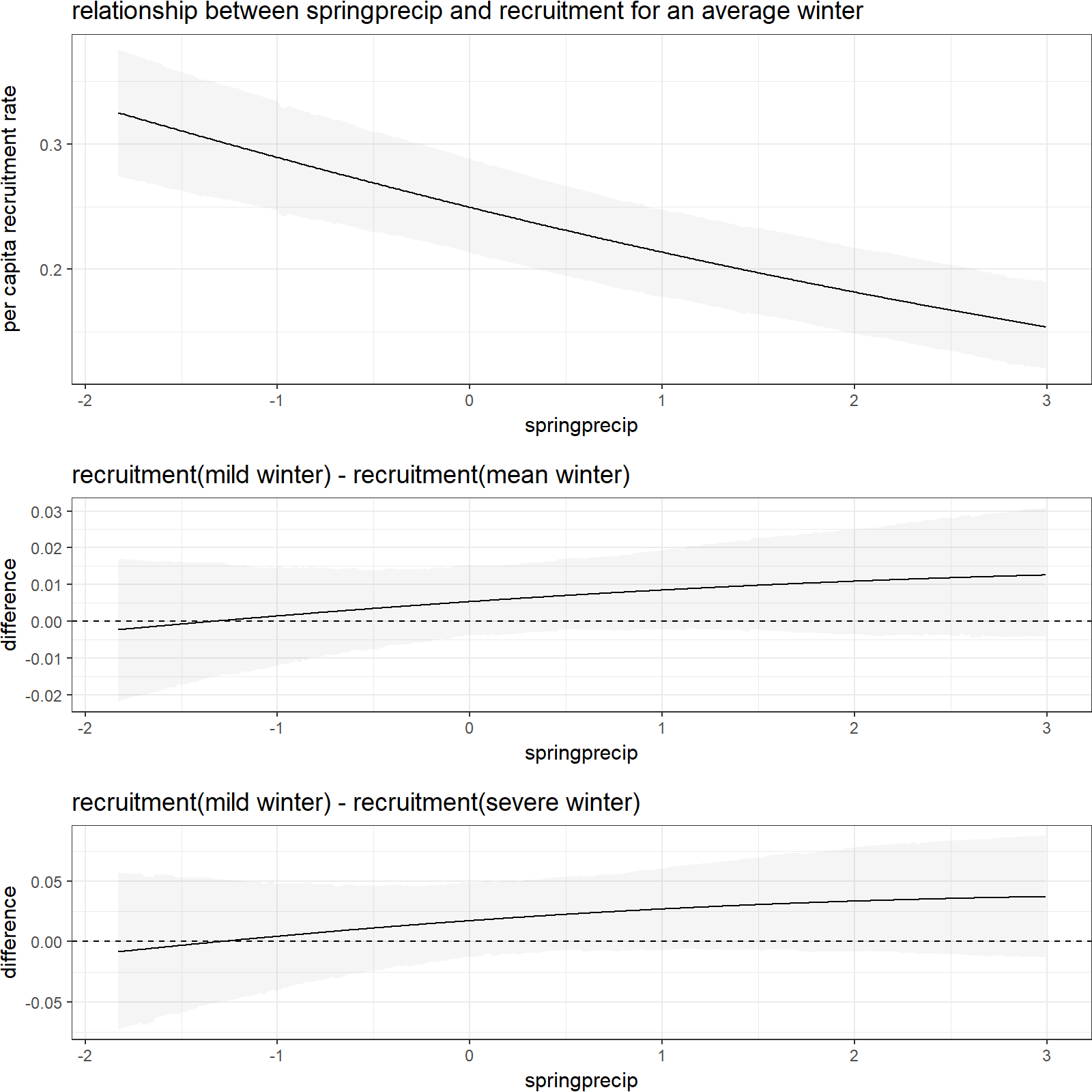

Supplement: S8 Fig — Spring precipitation was on a standardized scale, with 0 corresponding to the average (0.18 m) and 1 corresponding to one standard deviation above the average (0.22 m) (top panel). The second panel shows the predicted difference in recruitment rates between a mild winter (swe = 5th percentile of observed values) and a mean winter (swe = 0) as a function of spring precipitation, and the bottom panel shows the difference in recruitment rates between a mild winter and a severe winter (swe = 95th percentile of observed values). (TIF) [file pone.0226492.s008.tif]

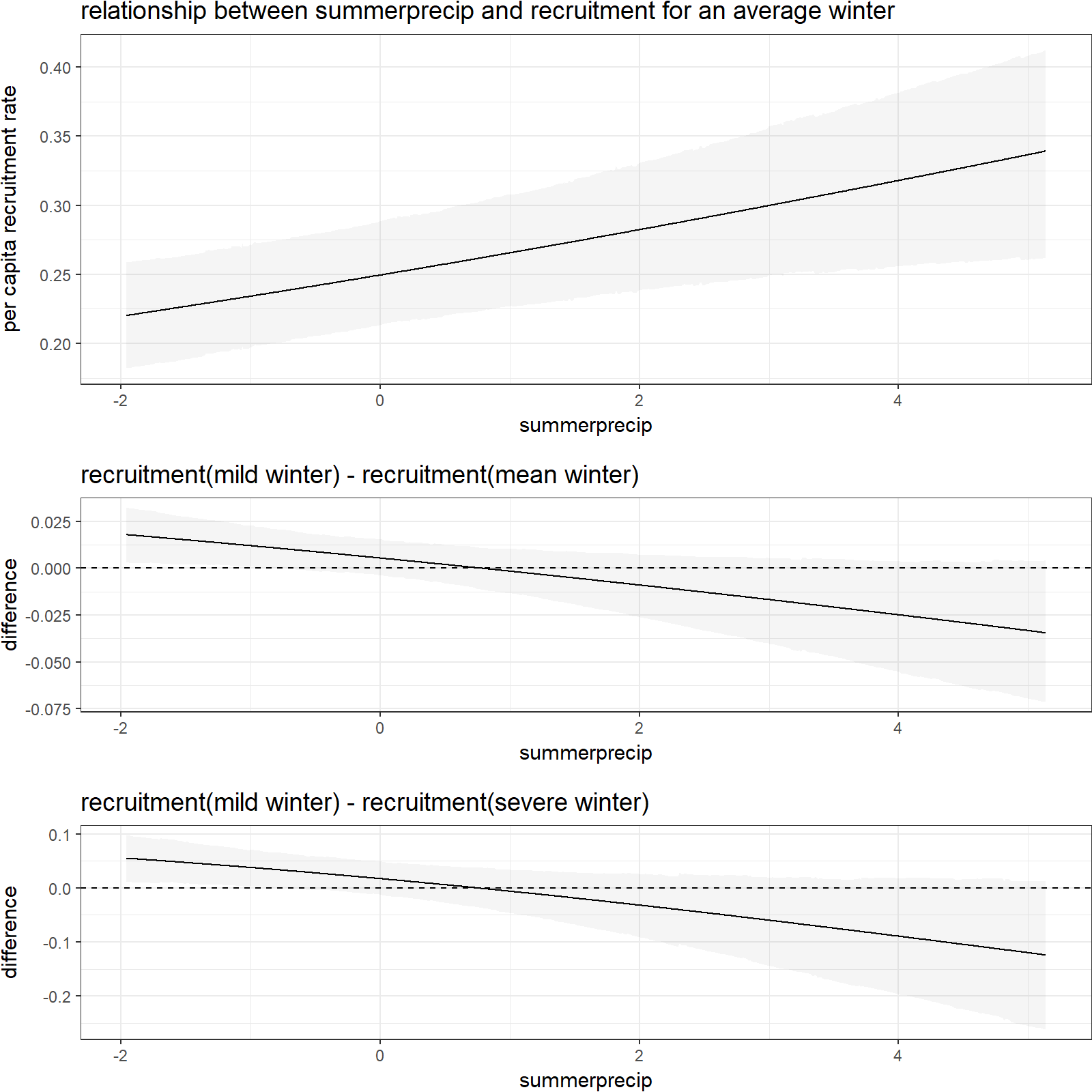

Supplement: S9 Fig — Summer precipitation was on a standardized scale, with 0 corresponding to the average (0.15 m) and 1 corresponding to one standard deviation above the average (0.19 m) (top panel). The second panel shows the predicted difference in recruitment rates between a mild winter (swe = 5th percentile of observed values) and a mean winter (swe = 0) as a function of summer precipitation, and the bottom panel shows the difference in recruitment rates between a mild winter and a severe winter (swe = 95th percentile of observed values). (TIF) [file pone.0226492.s009.tif]

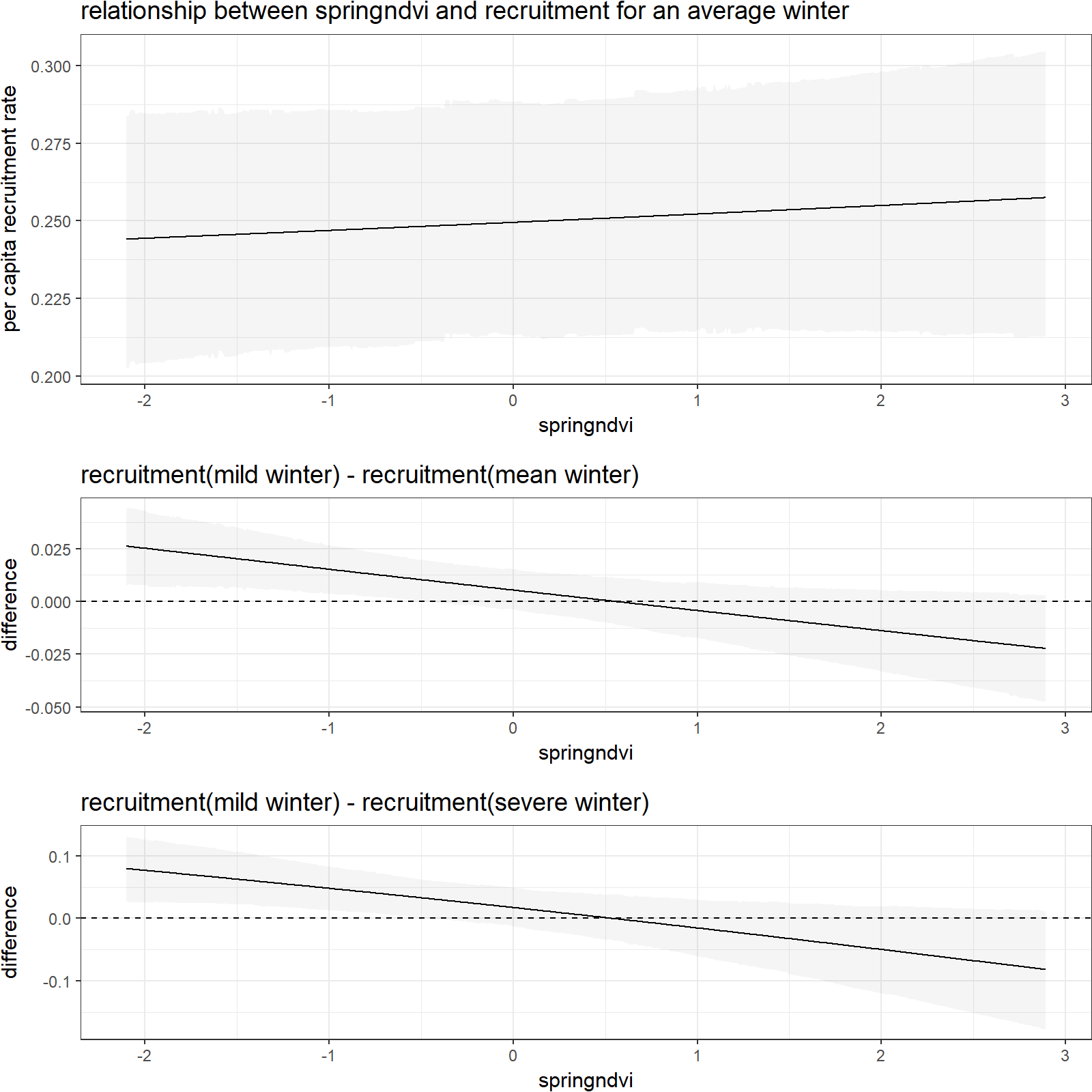

Supplement: S10 Fig — Spring NDVI was on a standardized scale, with 0 corresponding to the average (1.06) and 1 corresponding to one standard deviation above the average (1.40) (top panel). The second panel shows the predicted difference in recruitment rates between a mild winter (swe = 5th percentile of observed values) and a mean winter (swe = 0) as a function of spring NDVI, and the bottom panel shows the difference in recruitment rates between a mild winter and a severe winter (swe = 95th percentile of observed values). (TIF) [file pone.0226492.s010.tif]

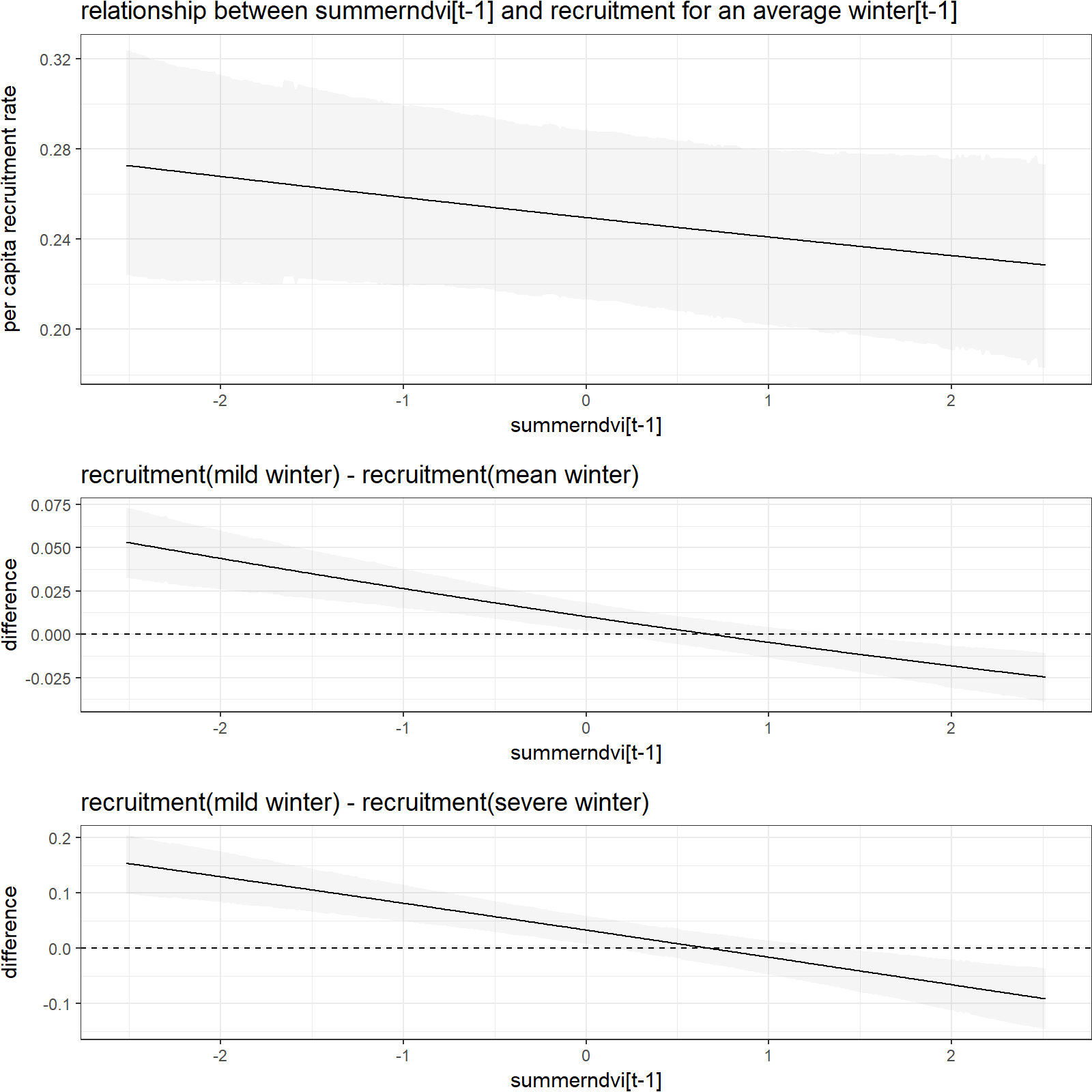

Supplement: S11 Fig — Summer NDVI was on a standardized scale, with 0 corresponding to the average (4.10) and 1 corresponding to one standard deviation above the average (4.85) (top panel). The second panel shows the predicted difference in recruitment rates between a mild winter (swe = 5th percentile of observed values) and a mean winter (swe = 0) as a function of lagged summer NDVI, and the bottom panel shows the difference in recruitment rates between a mild winter and a severe winter (swe = 95th percentile of observed values). (TIF) [file pone.0226492.s011.tif]
