# Supplementary material for: An improved understanding of ungulate population dynamics using count data: Insights from western Montana
Source: PLoS One. 2019 Dec 23;14(12):e0226492. doi: 10.1371/journal.pone.0226492 (PMC6927647; doi:10.1371/journal.pone.0226492)
Supplement: S1 Table — (DOCX) [file pone.0226492.s012.docx]

| covariate | mean | median | sd | 5% quantile | 95% quantile |
| --- | --- | --- | --- | --- | --- |
| lions | 4.12 | 3.00 | 3.86 | 0.00 | 11.00 |
| bears | 21.31 | 18.00 | 17.86 | 0.00 | 54.00 |
| wolves | 15.99 | 12.00 | 14.50 | 0.00 | 45.00 |
| spring ndvi | 1.10 | 1.07 | 0.38 | 0.53 | 1.79 |
| summer ndvi | 4.06 | 4.10 | 0.75 | 2.84 | 5.19 |
| spring precip | 0.17 | 0.16 | 0.05 | 0.11 | 0.26 |
| summer precip | 0.15 | 0.15 | 0.04 | 0.10 | 0.21 |
| swe | 8.15 | 6.27 | 6.30 | 1.84 | 21.27 |
