# Supplementary material for: An improved understanding of ungulate population dynamics using count data: Insights from western Montana
Source: PLoS One. 2019 Dec 23;14(12):e0226492. doi: 10.1371/journal.pone.0226492 (PMC6927647; doi:10.1371/journal.pone.0226492)
Supplement: S2 Table — (DOCX) [file pone.0226492.s013.docx]

| Parameter | median | 90% HPD interval | 50% HPD interval |
| --- | --- | --- | --- |
| bears | -0.046 | (-0.092, -0.003) | (-0.064, -0.028) |
| lions | -0.036 | (-0.073, -0.003) | (-0.05, -0.022) |
| wolves | 0.045 | (0.004, 0.088) | (0.029, 0.064) |
| springNDVI | 0.014 | (-0.027, 0.061) | (-0.004, 0.031) |
| summerNDVI | -0.047 | (-0.103, 0.014) | (-0.073, -0.025) |
| springPrecip | -0.203 | (-0.263, -0.143) | (-0.226, -0.177) |
| summerPrecip | 0.084 | (0.03, 0.134) | (0.064, 0.107) |
| swe | -0.030 | (-0.082, 0.024) | (-0.05, -0.006) |
| springPrecip*swe | -0.023 | (-0.064, 0.019) | (-0.04, -0.006) |
| summerPrecip*swe | 0.039 | (0.004, 0.074) | (0.026, 0.055) |
| springNDVI*swe | 0.054 | (0.011, 0.098) | (0.036, 0.072) |
| summerNDVI*swe | -0.029 | (-0.069, 0.01) | (-0.045, -0.013) |
| lions*swe | 0.020 | (-0.015, 0.055) | (0.005, 0.034) |
| bears*swe | -0.105 | (-0.164, -0.048) | (-0.128, -0.081) |
| wolves*swe | -0.065 | (-0.114, -0.018) | (-0.085, -0.046) |
| summerNDVI[t-1] | -0.047 | (-0.11, 0.016) | (-0.072, -0.022) |
| summerPrecip[t-1] | 0.047 | (-0.003, 0.097) | (0.023, 0.064) |
| swe[t-1] | -0.057 | (-0.101, -0.012) | (-0.075, -0.039) |
| summerPrecip*swe[t-1] | 0.026 | (-0.007, 0.06) | (0.011, 0.039) |
| summerNDVI*swe[t-1] | 0.084 | (0.053, 0.117) | (0.072, 0.098) |
