# Supplementary material for: An improved understanding of ungulate population dynamics using count data: Insights from western Montana
Source: PLoS One. 2019 Dec 23;14(12):e0226492. doi: 10.1371/journal.pone.0226492 (PMC6927647; doi:10.1371/journal.pone.0226492)
Supplement: S1 File — (DOCX) [file pone.0226492.s014.docx]

**Modeling**

We fit a single combination of covariates to the age ratio and population model that allowed for a variety of interactions between precipitation, primary production, predator metrics and winter severity.

For the population model, the response was the per capita recruitment rate in year $t$ and hunting district $u$ and covariates were modeled on the logit scale.

$$\mathrm{logit}\left( \tau_{t,u} \right)=\alpha+\zeta_{t}+\mathbf{X}_{t,u}B,$$

where $\alpha$ is a common intercept (and corresponds to mean recruitment on the logit scale), $\zeta_{t,u}$ are mean-zero random effects for year, and $\mathbf{X}_{t,u}$ is the vector of covariates and $\mathbf{B}$ the regression coefficients.

$$\mathbf{X}_{t,u}B=\beta_{lions}({lions harvested}_{t,u})+\beta_{bears}({black bears harvested}_{t,u})+\beta_{wolves}({wolf count}_{t,u})+\beta_{springNDVI}({spring NDVI}_{t,u})+\beta_{summerNDVI}({summer NDVI}_{t,u})+\beta_{springPrecip}({spring Precip}_{t,u})+\beta_{summerPrecip}({summer Precip}_{t,u})+\beta_{swe}(\mathrm{swe}_{t,u})+\beta_{springPrecip*swe}({spring Precip * swe}_{t,u})+\beta_{summerPrecip*swe}({summer Precip * swe}_{t,u})+\beta_{springNDVI*swe}({spring NDVI * swe}_{t,u})+\beta_{summerNDVI*swe}({summer NDVI * swe}_{t,u})+\beta_{lions*swe}({lions harvested*swe}_{t,u})+\beta_{bears*swe}({black bears harvested*swe}_{t,u})+\beta_{wolves*swe}({wolf count*swe}_{t,u})+\beta_{summerNDVI}({summer NDVI}_{t-1,u})+\beta_{summerPrecip}({summer Precip}_{t-1,u})+\beta_{swe}(\mathrm{swe}_{t-1,u})+\beta_{summerNDVI*swe}({summer NDVI*swe}_{t-1,u})+\beta_{summerPrecip*swe}({summer Precip*swe}_{t-1,u}).$$

We used the same covariates for the age ratio model, but note that the response uses the identity link (not logit). We included an additional covariate to try to account for the effects of female harvest on age ratios, $\beta_{harvest}$ (Lukacs et al. 2018).
